# LC-MS/MS-QTOF Screening and Identification of Phenolic Compounds from Australian Grown Herbs and Their Antioxidant Potential

**DOI:** 10.3390/antiox10111770

**Published:** 2021-11-05

**Authors:** Akhtar Ali, Yasmeen M. Bashmil, Jeremy J. Cottrell, Hafiz A. R. Suleria, Frank R. Dunshea

**Affiliations:** 1 School of Agriculture and Food, Faculty of Veterinary and Agricultural Sciences, The University of Melbourne, Parkville 3010, Australia; akali@student.unimelb.edu.au (A.A.); ybashmil@student.unimelb.edu.au (Y.M.B.); jcottrell@unimelb.edu.au (J.J.C.); hafiz.suleria@unimelb.edu.au (H.A.R.S.); 2 Faculty of Biological Sciences, The University of Leeds, Leeds LS2 9JT, UK

**Keywords:** herbs, medicinal plants, polyphenols, antioxidants, human health, LC-MS/MS

## Abstract

Culinary spices and herbs have been used to impart a characteristic flavour and aroma in food due to their appealing fragrance. Recently, bioactive compounds from herbs, especially phenolics, have gained much attention due to their potential health outcomes. The aim of this study was to characterize and quantify the phenolic compounds from 10 widely used Australian-grown herbs (oregano, rosemary, bay, basil, sage, fenugreek, dill, parsley, mint and thyme). For this purpose, liquid chromatography mass spectrometry (LC-MS) was used for the complete profiling of polyphenolic compounds and quantification of abundant phenolic compounds was completed with high-performance liquid chromatography—photodiode array detection (HPLC-PDA). Polyphenols from Australian-grown herbs were estimated through total phenolic content (TP), total flavonoids (TF) and total tannins (TT) along with their in-vitro antioxidant activities. Oregano and mint were estimated with the highest value of TP (140.59 ± 9.52 and 103.28 ± 8.08 mg GAE/g, milligram gallic acid equivalent/gram) while rosemary and mint had the highest TF (8.19 ± 0.74 and 7.05 ± 0.43 mg QE (quercetin equivalent)/g). In this study, eighty-four (84) phenolic compounds were screened and confirmed through LC-MS/MS by comparing their masses and fragmentation pattern with published libraries. The results of this study validate the use of these herbs as bioactives and their positive impact on human health.

## 1. Introduction

There is an increasing interest in culinary herbs and spices for scientific research and industry due to their potent antioxidant and antimicrobial properties. The use of culinary herbs and spices as a food ingredient has been a common practice since ancient times. Culinary spices and herbs are widely used to improve the food flavour because of their organoleptic, preservative and sensory attributes. Mediterranean herbs are widespread and grown in Australia on a large scale to fulfill the local consumption. Culinary herbs and spices are also used in industry as a food supplement or enrichment or fortification of different products such as snacks, biscuits, candies, pickles, syrups and jams due to their color, aroma and flavor, and therapeutic effects. Plant-based bioactives, particularly polyphenolic compounds, have gained much attention due to their positive health outcomes [1]. Many researchers reported culinary herbs and spices as a dietary source of natural antioxidant phenolic compounds, which ultimately has piqued other researchers’ interest in exploring the phenolic composition and antioxidant properties of widely used herbs and spices [2,3]. In the search of beneficial phytochemicals for human health, phenolic compounds are an attractive target, due to their potential health outcomes. Culinary herbs and spices have been used to treat various health conditions like bone fractures, aches, sprains, joint inflammation and healing of wounds. Furthermore, they are used in the food industry, feed industry, pharmaceutical and cosmetic industries. The broad utilization of herbs and spices has led to an upsurge the production worldwide. They are widely utilized as health-promoting ingredients for antidiabetic [4], anti-carcinogen, anti-hypertensive, anti-depression, anti-inflammatory [5], anti-HIV [6], antioxidant, antimicrobial, cardio- and neuroprotective agents and antipyretic [7,8,9,10,11] properties.

The bioactive compounds, mainly phenolics, are considered for their health-promoting aspects present in fruits, vegetables, herbs, spices and other medicinal plants and known as secondary metabolites in phytochemistry. The significant role of these metabolites in disease prevention and health promotion has been investigated and identified in recent research. Polyphenolic compounds, especially flavonoids and phenolic acids attracted the attention of food experts and nutritionists due to their potential health effects [12]. Nowadays, polyphenols are a target group to increase the shelf life of lipid-rich foods due to their potential antioxidant’s mechanism. 

Furthermore, antimicrobial phenolic constituents in herbs have food preservation characteristics. These bioactives have different functional properties including enzymatic activity, inhibition of cellular proliferation, free radical scavenging capacity, signal transduction pathways and metal chelation in the biological system. Free radicals potentially damage or alter the DNA biomolecules because of their high reactivity in the biological systems while endogenous antioxidants can minimize or neutralize the activity of these free radicals in the body [13]. Due to concerns associated with the side effects of synthetic antioxidants, the use of antioxidants naturally found in fruits, vegetables, herbs and spices gained much interest as an alternative and inexpensive to synthetic antioxidants in traditional and modern therapy. In recent times, many studies described a negative relationship between the use of herbs and spices and the prevalence of chronic diseases in humans. Herbs and spices contain bioactive compounds that positively affect the functioning of the human and animal bodies. The bioactive compounds include carotenoids, vitamins, and phenolic compounds such as phenolic acids, flavonoids, flavones, tannins, lignans and stilbenes. They have the potential to lessen the risk of cardiovascular diseases and various cancer [14].

The high concentration of free radicals in the body causes oxidative stress while natural antioxidants in culinary herbs and spices can reduce it. Chronic oxidative stress can cause different health ailments like cancers, cardiovascular diseases and especially accelerate the aging process. Therefore, culinary herbs and spices could be utilized to prevent the various health complications that originate from oxidative stresses and various metabolic disorders in the body [1]. Many studies have been conducted to identify, characterize, and explore the culinary herbs and spices due to their phenolic compounds and antioxidant activity. However, a comprehensive profiling of culinary herbs and spices is still missing due to their complex nature, structure, widespread cultivars at various geographical locations and mainly due to the unavailability of commercial standards for proper identification and validation of these bioactives in the system. An advanced analytical technique, LC-MS coupled with QTOF for the identification of unknown bioactive compounds from various plant samples including culinary spices and herbs.

The purpose of the current study was to investigate the commonly used Australian-grown herbs (rosemary, oregano, mint, thyme, fenugreek, parsley, dill, sage, bay and basil) for polyphenols and antioxidant potential. For this purpose, LC-MS/MS-quadrupole—time of flight (QTOF) was used to separate and identify the phenolic compounds (84) from the selected culinary herbs. Moreover, TP, TT, TF, 2,2′diphenyl-1-picrylhy-drazyl (DPPH), ferric reducing antioxidant power (FRAP), 2,2′-azinobis-(3-ethylbenzothiazoline-6-sulfonic acid (ABTS), ferrous ion chelating capacity (FICC), hydroxyl radical scavenging assay (**^∙^**OH−RSA), phosphomolybdate antioxidant power assay (PMAP) and reducing power assay (RPA) were performed for the estimation of total phenolics and their antioxidant activities. This study will provide a piece of useful and valuable information regarding the potent effects of herbal phenolic compounds on human health. Additionally, it will support the inclusion of culinary herbs and spices in different food and feed sectors as a potential bioactive constituent [15].

## 2. Materials and Methods

### 2.1. Materials

Analytical, HPLC and LCMS-grade chemicals were used in this study. Gallic acid, Folin–Ciocalteu reagent, vanillin, *L*-ascorbic acid, iron(III) chloride hexahydrate (Fe[III]Cl_3_•6H_2_O), sodium phosphate dibasic hepta-hydrate (Na_2_HPO_4_•7H_2_O), hexahydrate aluminium chloride sodium phosphate, sodium phosphate monobasic monohydrate (Na_2_HPO_4_•H_2_O), trichloroacetic acid (TCA), hydrochloric acid (HCl), ferrozine, hydrated sodium acetate, ethylene diamine tetra acetic acid (EDTA), potassium ferrocyanide (III), iron (II) and iron (III) chloride, 3-hydrobenzoic acid (C_7_H_6_O_3_), ammonium molybdate, iron (II) sulphate heptahydrate, catechin, quercetin, DPPH, 2,4,6-tripyridyl-s-triazine (TPTZ) and ABTS were purchased from the Sigma Aldrich (Castle Hill, Australia) for the estimation of polyphenols and antioxidant potential. Hydrogen peroxide (30%) and Sodium carbonate anhydrous were purchased from Chem-Supply Pty Ltd. (Adelaide, Australia) and 98% sulfuric acid was purchased from RCI Labscan (Rongmuang, Thailand). HPLC and LC-MS grade reagents include ethanol, methanol, acetonitrile, formic acid, glacial acetic acid and iron (III) chloride anhydrous were purchased from Thermo Fisher Scientific Inc (Scoresby, Australia). HPLC standards were also purchased from Sigma Aldrich.

### 2.2. Preparation and Extraction of Phenolic Compounds

Culinary herbs in powder or whole were bought from the local market in Victoria, Australia. The method of [1] was used for the extraction of phenolic compounds was as follows; 2 g sample and 30 mL of 80% methanol and 0.1% of formic acid in ultra-pure water was used for preparation of extracts from selected herbs in triplicate. The samples were then shaken in an orbital shaker (ZWYR-240 incubator shaker, Labwit, Ashwood, VIC, Australia) for 12 h at 4 °C and 150 rpm for possible extraction of phenolic compounds. Then, centrifugation of all samples was done with Hettich Refrigerated Centrifuge at 4 °C for 20 min at 8000 rpm. The supernatant was collected, filtered with the help of a 0.45-µm syringe filter. It was then stored at −20 °C for further analysis for a maximum period of seven days.

### 2.3. Antioxidant Activities

The estimation of antioxidant activities was carried out by following the previously established methods Ali, Wu, Ponnampalam, Cottrell, Dunshea and Suleria [1] while all tests were performed in triplicate.

### 2.4. LC-MS/MS Characterization of Phenolic Compounds

A previously developed method was used for the identification and complete mapping of the phenolic profile from selected herbs Suleria, et al. [16] while Agilent 6520 Accurate-Mass QTOF was applied in a positive and negative mode. Synergi Hydro-RP (4 μm particle size, 4.6 mm internal diameter and 250 mm length with 80 Å pore size was used for the separation of phenolic compounds, and the flow rate was set at 800 μL/min. An aliquot of 10 μL from each extract was injected while gradient was 0–5 min (0–10%), 5–25 min (10–25% B), 25–35 min (25–35% B), 35–45 min (40–60% B) 45–75 min (40–55% B), 75–80 min (55–88% B) (80–82 min (80–90% B), 82–85 min (90–100% B), 85–90 min (0% B). Mobile phase A was 0.1% formic acid in water and mobile phase B was 95% acetonitrile with 0.1% formic acid. A full scan mode was achieved in the range of 100–1000 amu with the following conditions; capillary voltage (3500 V), nozzle voltage (500 V), nitrogen gas flow rate (9 L/min) at 325 °C and nebulization was set as 45 psi while 10, 20 and 30 eV collision energies were used. MassHunter Workstation Software (LC/MS Data Acquisition for 6200 series TOF/6500 series Q-TOF) was used for extraction and identification of phenolic compounds.

### 2.5. HPLC-MS Semi-Quantification of Phenolic Compounds

Semi-quantification of polyphenolic compounds was achieved by following the method of [17] with some modifications by using the Water Alliance (2690) HPLC system equipped with Diode Array Detection (DAD-Model 2998). Briefly, 2% acetic acid in Milli-Q water was used as mobile phase A and acetic acid, water and acetonitrile were used as mobile phase B in the ratio of 0.5:49.5:50. An aliquot of 20 μL herbal extract was used for each injection with the following gradient profile; 0–20 min (90–75% A), 20–30 min (75–65% A), 30–40 min (65–60% A), 40–70 min (60–45% A), 70–75 min (45–20% A), 75–77 min (20–0% A), 79–82 min (0% A), 82–85 min (0–90% A). The flow rate was maintained at 800 μL/min while the temperature was unchecked. Twenty-eight (28) phenolic compounds were semi-quantified in this study.

### 2.6. Statistical Analysis

The data were submitted to one-way analysis of variance (ANOVA) followed by Tukey’s honestly significance test (HSD) at *p* ≤ 0.05 through Minitab Program for Windows version and 18.0. XLSTAT-2019.1.3 was used for correlation and Principal Component Analysis (PCA).

## 3. Results and Discussion

### 3.1. Polyphenols Estimation of Herbs

Polyphenolic compounds are plant-based phytochemicals. Nowadays, culinary herbs have gained more interest due to their potential bioactive compounds that may benefit human health. The data for the estimation of polyphenolic compounds are presented in Table 1.

TP was estimated by the Folin–Ciocalteu (F-C) method and oregano and mint exhibited the highest value of TP (140.59 ± 9.52 and 103.28 ± 8.08 mg GAE/g) while fenugreek with least value of TP (7.58 ± 0.35 mg GAE/g). Previously, TP value of oregano (101.7 mg GAE/g), mint (51.5 mg GAE/g), basil (36.4 mg GAE/g), rosemary (50.7 mg GAE/g), sage (53.2 mg GAE/g), thyme (45.2 mg GAE/g) and bay (41.7 mg GAE/g) respectively also showed the same trend reported by Shan, et al. [18]. Previously, Słowianek and Leszczyńska [19] also studied parsley, basil, oregano and thyme who found 13.6, 26.5, 51.3 and 71.7 mg GAE/g total polyphenols, respectively. The higher values of TP for oregano, mint and rosemary indicate that solvent ethanol (70%) with 0.1% formic acid performed the better extraction or might be due to different herbal cultivars were used in current and latter study for extraction and quantification of phenolic contents. TP values indicates the diverse nature of phenolic compounds in Australian-grown herbs.

Moreover, the highest TF value was found in rosemary (8.19 ± 0.74 mg QE/g), mint (7.05 ± 0.43 mg QE/g) and sage (6.05 ± 0.76 mg QE/g). On the other hand, the least value of TF was found in fenugreek (1.57 ± 0.16 mg QE/g) and parsley (1.51 ± 0.13 mg QE/g), respectively. Overall, flavonoids are more abundant in compounds than other phenolics in herbs. In contrast, TT value of mint (8.31 ± 1.58 mg QE/g) and basil (6.16 ± 0.31 mg QE/g) were found higher than other herbs. Fenugreek was found with the least TT value (3.46 ± 0.20 mg QE/g). Flavonoids and tannins are the important groups of phenolic compounds. Therefore, the screening and identification of these bioactive compounds from herbs with LC-MS-MS-QTOF is important because it can deliver much more reliable and authentic data for the application of these bioactives in human food to treat various ailments.

### 3.2. Antioxidant Activities of Herbs

The radical scavenging capacity of Australian-grown herbs were estimated by ABTS, RPA, DPPH, FRAP, PMAP, FICC and ^•^OH−RSA (Table 2). Fruits, vegetables, herbs and spices are used as a source of antioxidant chemical constituents in the human diet capable of deactivating the free radicals. Generally, phenolic compounds are considered the dynamic antioxidant constituents in culinary herbs and spices that have different health effects. They are considered as versatile compounds in plants that act as anti-radicals, metal chelators, reducing agents and hydrogen ion donators [1]. Moreover, the antioxidant potential is expressed as a parameter to indicate the benefits of culinary herbs and spices consumed as food. There are a variety of substances in herbal plants that have been suggested to serve as antioxidants. Different bioactive compounds, especially phenolics such as phenolic acids, flavonoids, coumarins, tannins, xanthones, lignans, stilbenes, other polyphenols and more recently procyanidins are reported as promising therapeutic agents to scavenge free radicals [20].

DPPH is a low-cost assay frequently used to estimate the ability of samples to scavenge free radicals in the biological system based on electron or hydrogen ion donation ability [21]. DPPH˙ is a free radical with a stable centered nitrogen, which reduces its bluish-purple color when mixed with herbs extract [2]. Any substance that causes this reaction can be classified as an antioxidant; thus, they are known as radical scavengers [2]. From Table 1, DPPH values of rosemary (25.09 ± 0.67 mg AAE/g), oregano (23.24 ± 1.23 mg AAE/g), mint (21.65 ± 0.36 mg AAE/g) and sage (21.43 ± 0.51 mg AAE/g) were estimated higher (*p* < 0.05) than other listed herbs. Previously, the higher value of DPPH for rosemary than oregano, thyme and bay had been reported in some studies [3,22]. The higher value of flavonoids in rosemary could be the reason for the higher DPPH value. Many studies reported that rosemary and oregano had higher radical scavenging capacity than other herbs [3,19,22].

The RPA values of rosemary (37.20 ± 3.85 mg AAE/g), bay (18.78 ± 4.73 mg AAE/g) oregano (16.98 ± 1.34 mg AAE/g) were estimated higher than other listed herbs while the least RPA value (4.22 ± 0.13 mg AAE/g) found in fenugreek. The RPA could provide a significant indication of the antioxidant potential of the herbal extracts. The iron (III) reduction in RPA is often used as an indicator for the mechanism of phenolic compounds antioxidant reaction through which they donate electrons [2]. The reductones in herbal extracts are responsible for the reducing properties of herbs. They reduce Fe^3+^—ferricyanide complex to the ferrous form during reaction. Generally, reductones break the free radical chain by donating a hydrogen atom or preventing peroxide formation by reacting with certain peroxide precursors [23,24].

The iron chelation ability of extracts depends on the function group of iron used in the biological system. In this quest, rosemary and thyme were found with higher FICC values (1.68 ± 0.04 and 1.35 ± 0.08 mg EDTA/g, respectively) than other listed herbs. The FICC of an herbal compound is vital because it reduces the concentration of transition metals during lipid peroxidation responsible for catalyzing. The chelating agents (secondary antioxidants) reduce the redox potential by stabilizing metal ions’ oxidized form after forming s-bonds with a metal during reaction [23,25]. It is believed that ferrous ion via the Fenton’s reaction increased lipid peroxidation through dismantling the hydrogen and lipid peroxides to free radicals. The lipid peroxidation also increased when ferrous ion decomposed lipid hydroperoxides into alkoxyl and peroxyl radicals. In this assay, ferrozine forms complex bond with ferrous ion while the herbal extracts inhibit the complex formation. Thus, herbal extracts provide protection against oxidative damage by minimizing the ferrous ions.

^•^OH−RSA was also used to determine the anti-radical capacity of herbs. The higher value of ^•^OH−RSA was estimated in rosemary (26.09 ± 1.73 mg AAE/g) while the least value was estimated in fenugreek (3.19 ± 0.29 mg AAE/g). Hydroxyl radicals (^•^OH) are the most reactive species that cause lipid peroxidation, DNA damage, and enormous biological damage by attacking almost every molecule found in the biological system [26]. Consequently, the scavenging of ^•^OH radicals by herbal extracts could provide a significant protection to biological damage against these free radicals. Phosphomolybdenum antioxidative power assay (PMAP assay) is used to measure the reduction capacity of molybdenum (VI) to molybdenum (V) by an antioxidant phenolic compound and subsequent formation of a green molybdenum (V)/phosphate complex [27,28]. The results of PMAP assay indicate that rosemary and bay have significantly higher total antioxidant activity (21.93 ± 2.82 and 18.53 ± 0.16 mg AAE/g, respectively) than other listed herbs [27,28].

### 3.3. LC-MS/MS Screening and Identification of Polyphenolic Compounds

The untargeted screening and identification of polyphenols from Australian herbs were conducted through LC-MS/MS-QTOF (Table 3). The base peak chromatogram of each sample in positive and negative mode given in Figure S1, while chromatogram and spectrum of some selected compounds with their observed mass and retention time given in Figure S2. 

Twenty-eight (28) phenolic acids, thirty-seven (37) flavonoids, thirteen (13) other polyphenols, one (1) stilbene and five (5) lignans were identified using fragmentation patterns observed in MS/MS-QTOF mass spectra (Table 3). Due to the increasing interest and awareness about the antioxidant potential and associated health benefits of phenolic compounds, particularly herbs have gained much attention from nutritionists and food scientists.

#### 3.3.1. Phenolic Acids

**Figure 1:**
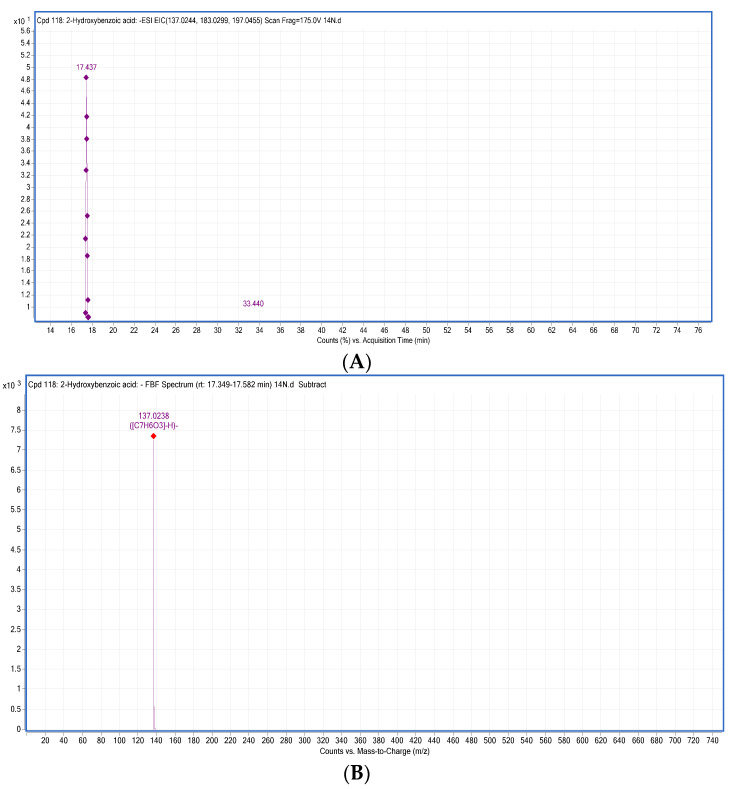

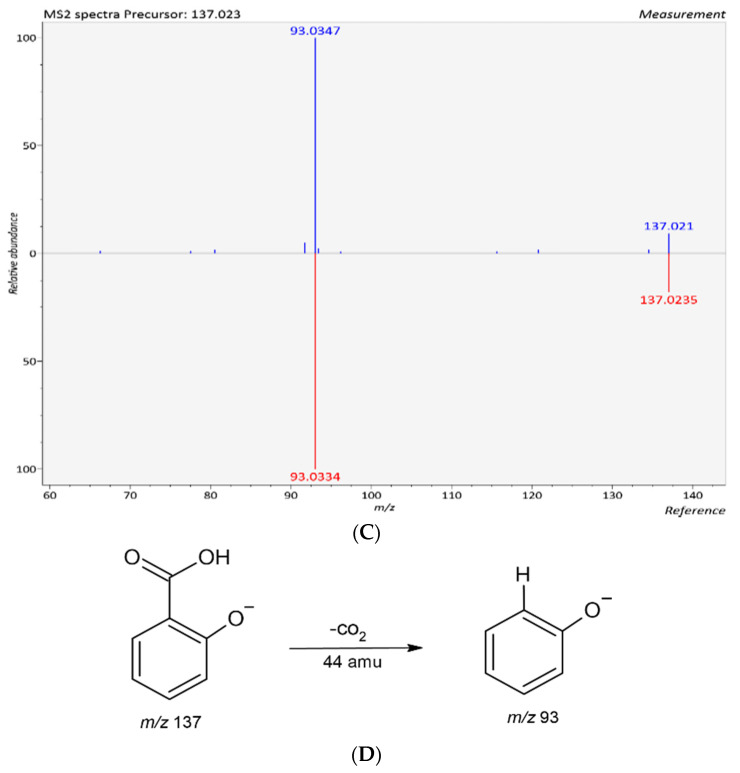
LC-MS/MS screening of 2-hydroxybenzoic acid (compound **8**). A chromatogram (**A**) and a mass spectrum (**B**) of 2-hydroxybenzoic acid are presented, which was identified from thyme (sample code 14N) in negative mode while MS/MS product ion spectra (**C**) was confirmed through online LC-MS library and database; (**D**) a possible fragmentation pattern of 2-hydroxybenzoic acid in negative mode is also presented with the loss of CO_2_ (44 amu).

They are widespread aromatic secondary plant metabolites and have promising health effects. A total of 28 phenolic acids (16 hydroxycinnamic acids, eight hydroxybenzoic acids, two hydroxyphenylpropanoic acids, one hydroxyphenyl pentatonic acid and one hydroxyphenyl acetic acids) were tentatively characterized and MS/MS used for the confirmation of their fragmentation pattern. Mainly, phenolic acids show the fragmentation pattern through the removal of CO_2_ and hexosyl moiety from the parent ions [1]. The compounds **1** (gallic acid), **5** (protocatechuic acid 4-*O*-glucoside), **6** (2,3-dihydroxybenzoic acid) and **8** (2-hydroxybenzoic acid, Figure 1) exhibited the product ions at *m*/*z* 125, *m*/*z* 153 *m*/*z* 109 and *m*/*z* 93 with the neutral loss of CO_2_ (44 Da) from the parent ions, respectively.

4-hydroxybenzoic acid 4-*O*-glucoside (compound **7**—C_13_H_16_O_8_) was detected at 299.0774 which generated daughter ions at *m*/*z* 255 and 137 via the removal of CO_2_ and hexosyl moiety from the parent ion, respectively while gallic acid 4-*O*-glucoside (compound **3**) exhibited the fragment ions at *m*/*z* 169 (gallic acid) and *m*/*z* 125 via the loss of hexosyl moiety (162 Da) and CO_2_ (44 Da) from the parent and fragment ions, respectively [29]. Yisimayili, et al. [30] had also stated gallic acid 4-*O*-glucoside in their studies. Gallic acid is one of the most abundant phytochemicals, which has potent anti-inflammatory, anti-mutagenic, antioxidant and anti-carcinogenic properties [31].

Sixteen compounds were identified in the class of hydroxycinnamic acids which makes them more abundant than other phenolic acids. Caffeoyl glucose (compound **9**) was characterized at *m*/*z* 341.0877 which produced daughter ions at *m*/*z* 179 (caffeic acid) and *m*/*z* 161 with the loss of two hexosyl moieties (162 Da) and one water unit (H_2_O—18 Da) from the precursor ion, respectively. Caffeoyl tartaric acid (compound **10**) was detected in mint and basil at *m*/*z* 311.0401 which produced one fragment ion at *m*/*z* 161 via the removal of 150 amu due to tartaric acid fission supported by LC-MS/MS. The compound **14** (3-feruloylquinic acid) was characterized in mint, generating daughter ions at *m*/*z* 298, 288, 192 and 191, respectively. *m*-coumaric acid (163.0405) was identified in basil and rosemary, which produced a fragment ion at *m*/*z* 119 via the removal of CO_2_ unit. Some studies had also reported coumaric acid in rosemary [22].

The most important phenolic compound of herbs (rosmarinic acid) was identified in rosemary, thyme, mint, oregano, basil and bay in negative mode. It is endowed by various potent health properties like antioxidant, anti-inflammatory, antiulcerogenic and anti-depressant [32]. It produced the fragment ions at *m*/*z* 179 (caffeic acid) and two caffeic acid fragments at *m*/*z* 161 and 135 via the removal of water unit (18 Da) and one unit of CO_2_ moiety, respectively from the daughter ion [33,34]. Caffeic acid (compound **19**, 179.0353) was also identified in almost all herbs which produced fragment ions at *m*/*z* 161 and 135 via the removal of water unit (H_2_O) and CO_2_. The compound **24** (cinnamyl glucose) was tentatively identified at *m*/*z* 309.0995 that generated the fragment ions at *m*/*z* 147, 131 and 103 through the loss of one hexosyl moiety (162), C_6_H_10_O_6_ (178 Da) and C_7_H_10_O_7_, respectively [1].

#### 3.3.2. Flavonoids

Flavonoids are also important secondary metabolites and indispensable components of nutraceutical, functional, medicinal and pharmaceutical applications. They have potent antioxidant, anti-mutagenic, anti-carcinogenic and anti-inflammatory properties [35]. In this quest, a total of 36 flavonoids were identified in selected herbs. (+)-Gallocatechin

(Compound **29**, C_15_H_14_O_7_) was tentatively identified at *m*/*z* 305.0660, which formed the fragment ions at *m*/*z* 261 and 219 via the removal of one unit of CO_2_ (44 amu) and one unit of C_3_O_2_ (86 amu) from the precursor ion, respectively [36]. It is commonly found in tea, red wine and cocoa, etc. They are well known for their antioxidant and cardiovascular protective effects [37]. Myricetin 3-*O*-rhamnoside (compound **46**—*m*/*z* 463.0872) was detected, which generated a daughter ion at *m*/*z* 317 via the loss of rhamnoside moiety from the parent ion. Quercetin 3′-*O*-glucuronide (compound **47**—477.0676) and quercetin 3-*O*-arabinoside (compound **50**—433.0783) were formed the fragment ions at *m*/*z* 301 via the subtraction of one unit of glucuronide (176 Da) and one unit of C_5_H_8_O_4_ (132 Da) from the precursor ions, respectively. Previously, these were reported in mint and lemon with strong antioxidant potential [38,39]. The compound **52** (3,7-Dimethylquercetin—C_17_H_14_O_7_) was detected in basil, mint, oregano, rosemary and sage in positive and negative modes, which produced daughter ions at *m*/*z* 314, 299, 217 through the removal of CH_3_, 2CH_3_ and two CH_3_ plus one unit of CO [40,41].

#### 3.3.3. Other polyphenols

Four (04) hydroxycoumarins, one (01) hydroxybenzoketones, two (02) tyrosols, three (03) phenolic terpenes, one (01) alkylphenols and two (02) other polyphenols were also identified (Table 3). The compound **69** (umbelliferone) was tentatively characterized at *m*/*z* 161.0242 in both modes, which produced fragment ions at *m*/*z* 133, *m*/*z* 117 and *m*/*z* 105 via the neutral loss of CO (28 Da), CO_2_ (44 Da) and C_2_H_2_ (28 Da) from the parent and former daughter ion, respectively [42]. The compound **71** (oleoside 11-methylester) was identified in negative mode at *m*/*z* 403.1238 which produced fragment ions at *m*/*z* 223 and *m*/*z* 165 via the neutral loss of glycoside (180 Da), and glycoside moiety and methyl ester (238 Da) from the parent ion confirmed through MS/MS. The compounds, rosmanol (C_20_H_26_O_5_—*m*/*z* 345.1693), carnosol (C_20_H_26_O_4_—*m*/*z* 329.1742) and carnosic acid (C_20_H_28_O_4_—331.1911) were produced fragment ions at *m*/*z* 301, *m*/*z* 285 and *m*/*z* 287, respectively via the removal of CO_2_ from the parent ions. Carnosic acid was previously reported in cinnamon, thyme, oregano and rosemary [22]. A recently published study also reported rosmanol, carnosol and carnosic acid in rosemary with significant antioxidant potential [43].

#### 3.3.4. Lignans and Stilbenes

Lignans (the bioactive compounds) characterized through LC-MS/MS have substantial anticarcinogenic, anti-inflammatory and antioxidant properties. Compound **79** in [M + H]^+^ mode at *m*/*z* 299.1268 was identified as enterolactone that showed fragments at *m*/*z* 281, *m*/*z* 187 and *m*/*z* 165 with the neutral loss of H_2_O (18 Da), C_6_H_8_O_2_ (112 Da) and C_9_H_8_O_2_ (148 Da), respectively [44]. Piceatannol (compound **84**) was characterized at *m*/*z* 243.0662 in negative mode generating daughter ions at *m*/*z* 255, 201, 175 and 159 via the removal of H_2_O, C_2_H_2_O from the precursor ion [45]. Piceatannol is a stilbene with two phenol rings and is extensively studied in grapes and red wine with significant health effects as antioxidant, anti-cancer, anti-mutagenic, anti-atherosclerotic and anti-inflammatory [46,47].

The application of LC-MS/MS-QTOF to Australian-grown herbs had allowed us to screen and detect 84 polyphenolic compounds with their product ions. No single study had been conducted yet to characterize all these Australian-grown herbs for their phenolic compounds. The screening of these bioactive constituents in these selected herbs can establish a new science to understand their potent health benefits. There is a considerable potential to identify novel unknown bioactive compounds by using this advanced analytical approach. One limitation of low collision energies is that they could not localize the position of native phenolic ring in the study of LC-MS/MS that went under modification. To solve this limitation, Nuclear magnetic resonance (NMR) would be beneficial because it can identify the responsible compounds for modification.

### 3.4. HPLC-MS Semi-Quantification of Phenolics from Herbs

**Figure 2:**
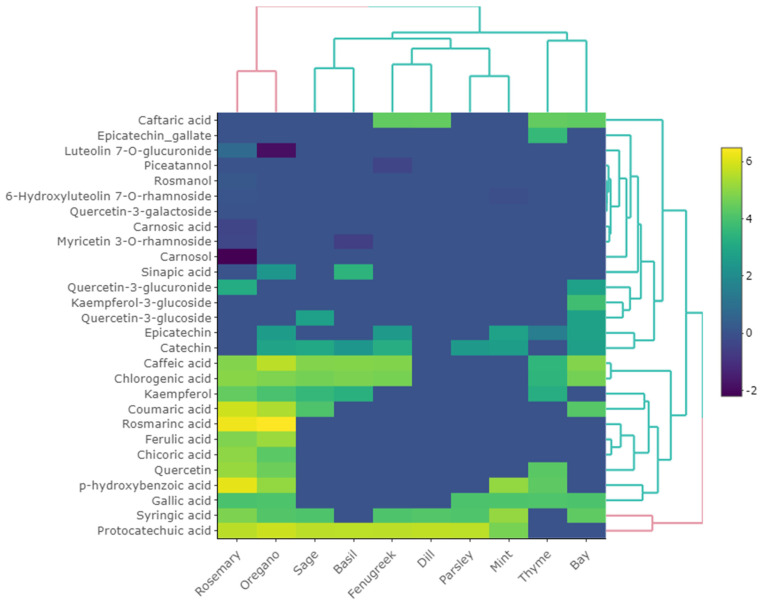
The distribution and concentration of polyphenolic compounds is represented through heatmap where yellow color indicates higher concentration while green and blue color indicates lower or zero concentration.

Phenolic compounds were mapped through HPLC-MS and heatmap shows the row and column-wise hierarchical clustering (Figure 2). The difference in clustering and color profile variation indicates the abundance of quantified polyphenolic compounds in 10 selected herbs.

Overall, rosemary and oregano were identified with a higher concentration of phenolic compounds (Table S1). Mainly, phenolic acids, flavonoids, phenolic terpenes and stilbenoids are included in the quantified phenolics. The main and most abundant phenolic compound identified in oregano was rosmarinic acid (1650.13 ug/g) while also quantified in rosemary (540.76 μg/g), thyme (210.23 μg/g), mint (199.54 μg/g), basil (167.84 μg/g) and bay (154.34 μg/g). Along with rosmarinic acid, rosmanol (1.17 μg/g), carnosol (0.11 μg/g), and carnosic acid (0.65 μg/g) were quantified in rosemary. These compounds have potent antioxidant activities [22]. Previously, Shan, Cai, Sun and Corke [18] also quantified rosmarinic acid in oregano (2562.7 mg/100g DW), rosemary, mint, thyme and basil. Gallic acid also quantified in the range of 53–58 μg/g in oregano, mint, thyme, rosemary, bay and parsley. The highest amount of protocatechuic acid quantified in oregano (331.45 μg/g) while the lowest amount was found in mint (110.01 μg/g). The highest amount of *m*-coumaric acid and 2-hydroxybenzoic acid were quantified in rosemary (340.44 μg/g) and oregano (230.07 μg/g), respectively. Moreover, the highest amount of chlorogenic acid and caffeic acid was quantified in rosemary (140.05 μg/g) and oregano (270.05 μg/g), respectively. Caffeic acid was quantified in the range of 32.12—270.05 μg/g in oregano, basil, bay, thyme, rosemary and sage. The lowest amount of caffeic acid was quantified in thyme (32.12 μg/g). In another study, caffeic acid and derivatives and syringic acid were quantified in rosemary [48,49] while chlorogenic acid and caffeic acid were quantified in sage and rosemary in a separate study [50].

The highest amount of catechin were quantified in fenugreek (24.60 μg/g) while the lowest was quantified in basil (10.64 μg/g). On the other hand, epicatechin gallate was only quantified in thyme (36.67 μg/g). Quercetin-3-glucuronide was quantified in rosemary (23.12 μg/g) and bay (14.86 μg/g), while quercetin-3-glucoside was quantified in bay (15.28 μg/g) and sage (14.65 μg/g). The highest amount of quercetin was quantified in rosemary (170.95 μg/g), while the lowest amount was found in thyme (70.30 μg/g). Luteolin 7-*O*-glucuronide and 6-hydroxyluteolin 7-*O*-rhamnoside were also quantified in rosemary in the range of 1.07–2.13 μg/g. Myricetin 3-*O*-rhamnoside was also quantified in mint (0.55 μg/g) and rosemary (0.69 μg/g), respectively. Piceatannol was quantified in fenugreek (0.65 μg/g), while kaempferol were quantified in the range of 26.95–81.55 μg/g in oregano, rosemary, thyme, basil and sage. Previously, gallic acid, caffeic acid, protocatechuic acid, coumaric acid, rosmarinic acid, carnosol, carnosic acid, kaempferol, catechin and other flavonoids were quantified in different herbs [18] while Vallverdú-Queralt, Regueiro, Martínez-Huélamo, Alvarenga, Leal and Lamuela-Raventos [22] also quantified caffeic acid, catechin, chlorogenic acid, epicatechin, ferulic acid, coumaric acid, *p*-hydroxybenzoic acid, protocatechuic acid, rosmarinic acid, syringic acid and quercetin in their study. Overall, clustering indicates that rosemary, oregano, sage, bay and thyme have higher concentrations than other listed herbs.

### 3.5. Pearson’s Correlation among Polyphenolics and Their Antioxidant Activities

For correlation purposes, Pearson’s correlation was conducted between the results of antioxidant assays and quantified phenolics to understand the possible behavior of herbs (Table 4).

It had been reported that total phenolics and total flavonoids are more responsible for antioxidant activities than other classes due to their abundance. Since phenolic compounds are vital antioxidant agents in herbs, we investigated TP, TF, and TT in 10 herbs. The value of TP was found in the range of 7.58 to 140.59 mg GAE/g while the average TP value was calculated as 50.36 mg GAE/g (Table 1).

Highly significant correlation (*p* ≤ 0.01) was observed between TF and antioxidant activities (DPPH, FRAP, ABTS, FICC and •OH−RSA) while TP correlated with DPPH, FRAP and ABTS with r^2^ = 0.721 (*p* ≤ 0.05), r^2^ = 0.576 (*p* ≥ 0.05) and r^2^ = 0.866 (*p* ≤ 0.01). Interestingly, a negative correlation was found between TT and antioxidant activities. Previously, Kam et al. [51] also reported that tannins have a limited antioxidant potential than flavonoids [18,52,53]. DPPH was highly correlated with FRAP, ABTS •OH−RSA while FRAP was highly correlated with ABTS, RPA, •OH−RSA, FICC, phenolic acids, flavonoids, phenolic terpenes and stilbenoids.

**Figure 3:**
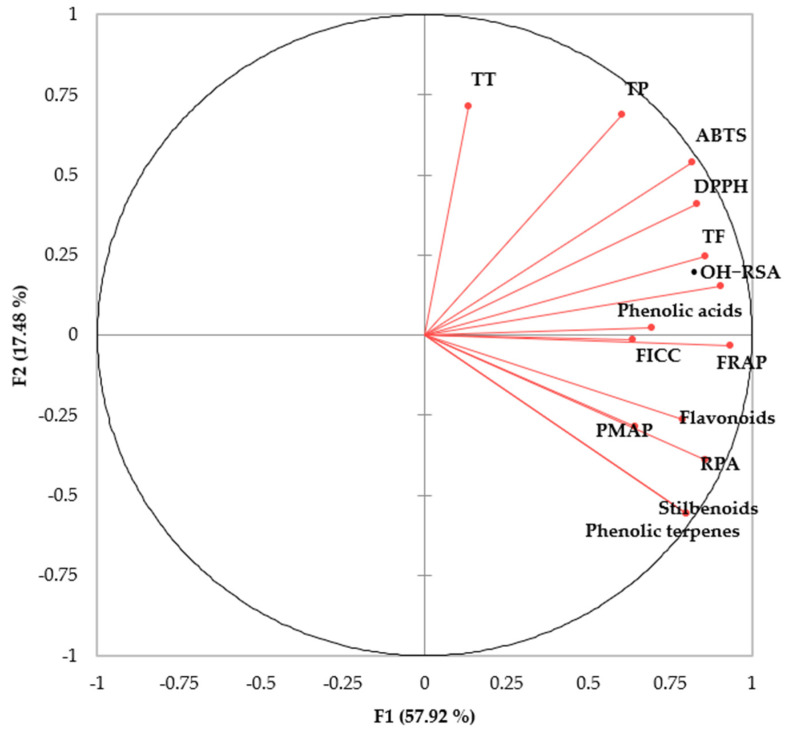
Principal component analysis (PCA) of the polyphenolic contents (TP, TF, TT, phenolic acids and flavonoids) and their antioxidant capacities (DPPH, ABTS, FRAP, RPA, •OH−RSA, PMAP and FICC) of 10 selected herbs.

PCA (Figure 3) clearly demonstrates that phenolic acids and flavonoids have a positive correlation with antioxidant activities. Overall, PCA shows the diversity of bioactive compounds. Total tannin compounds have a negative correlation, meaning their overall contribution in antioxidant activities are limited. PCA shows that phenolic acids are positively correlated with total polyphenols, which means that phenolic acids are abundant phenolic compounds in herbs which is accordance to previously published research [18]. Same trend was reported by Kim, et al. [54] where they found that antioxidant activities mainly depend on phenolic acids and flavonoids. Previously, polyphenolic compounds and antioxidant activities from herbs and spices were positively correlated by Lu, Yuan, Zeng and Chen [53].

After analyzing the results, it is suggested that non-phenolic constituents in herbs may also contribute towards antioxidant properties. Antioxidant activities depends on phenolic structure, synergistic and antagonistic action and concentration of these bioactives in the biological system. Furthermore, LC-MS/MS-QQQ can deliver more reliable data for quantifying these bioactive polyphenolic compounds, which will lead to understand the relationship among polyphenolics, structure and antioxidant properties.

## 4. Conclusions

In conclusion, Australian-grown herbs have a considerable number of polyphenolic compounds with significant health potential. The results in our study disclosed that herbs have reducing, and free-radical scavenging properties while phenolic acids and flavonoids are significantly contributing for antioxidant activities. In this study, a total of 84 polyphenolic bioactive compounds were screened and confirmed through LC-MS/MS-QTOF. The screening of these compounds will provide a significant contribution to the application of these bioactives for human health. The everyday use of these herbs could make a considerable contribution of human health and nutrient uptake. Owning to extended anti-radical properties of these herbs, their usage is endorsed in animal feed, human food and the nutraceutical and pharmaceutical industries. Further work should be conducted to determine the bioavailability of these compound if they are to be used for their medicinal and nutritional attributes. Cell-culture and in vivo studies have to be directed to assess their bioaccessibility and bioavailability of these secondary metabolites for commercial purposes.

## Figures and Tables

**Table 1:** Total phenolic content, total flavonoids and total tannins of 10 widely used herbs.

Herbs	TP (mg GAE/g)	TF (mg QE/g)	TT (mg CE/g)
Oregano	140.59 ± 9.52 ^a^	5.15 ± 0.12 ^bc^	5.23 ± 0.50 ^c^
Mint	103.28 ± 8.08 ^b^	7.05 ± 0.43 ^ab^	8.31 ± 1.58 ^a^
Thyme	43.16 ± 1.54 ^d^	4.30 ± 0.26 ^c^	4.93 ± 0.26 ^d^
Basil	39.91 ± 1.39 ^de^	3.62 ± 0.16 ^d^	6.16 ± 0.31 ^b^
Rosemary	58.66 ± 1.68 ^c^	8.19 ± 0.74 ^a^	4.69 ± 0.23 ^d^
Bay	33.93 ± 2.07 ^e^	1.84 ± 0.38 ^e^	6.09 ± 0.14 ^b^
Sage	47.62 ± 2.03 ^d^	6.05 ± 0.76 ^b^	4.41 ± 0.43 ^d^
Dill	16.41 ± 0.61 ^f^	3.70 ± 0.23 ^d^	5.28 ± 0.65 ^c^
Parsley	12.43 ± 3.20 ^g^	1.51 ± 0.13 ^e^	5.14 ± 0.68 ^c^
Fenugreek	7.58 ± 0.35 ^h^	1.57 ± 0.16 ^e^	3.46 ± 0.20 ^e^

Results are reported on dry weight basis in triplicate; the values with significant difference (*p* ≤ 0.05) are indicated by superscript letters (^a–h^).

**Table 2:** Antioxidant activities of 10 widely used herbs.

Herbs	DPPH (mg AAE/g)	FRAP (mg AAE/g)	ABTS (mg AAE/g)	RPA (mg AAE/g)	FICC (mg EDTA/g)	^•^OH−RSA (mg AAE/g)	PMAP (mg AAE/g)
Oregano	23.24 ± 1.23 ^ab^	10.72 ± 1.44 ^b^	111.12 ± 2.81 ^a^	16.98 ± 1.34 ^b^	0.73 ± 0.08 ^c^	17.72 ± 0.35 ^b^	10.06 ± 0.21 ^c^
Mint	21.65 ± 0.36 ^ab^	6.91 ± 0.77 ^c^	106.99 ± 2.90 ^ab^	8.61 ± 3.16 ^d^	1.22 ± 0.04 ^b^	16.22 ± 0.16 ^b^	12.82 ± 0.28 ^c^
Thyme	18.71 ± 0.52 ^b^	3.45 ± 1.67 ^d^	69.27 ± 0.56 ^d^	10.05 ± 0.88 ^cd^	1.35 ± 0.08 ^b^	14.74 ± 0.23 ^b^	7.93 ± 0.14 ^d^
Basil	18.64 ± 0.38 ^b^	7.56 ± 5.15 ^c^	65.73 ± 2.38 ^d^	8.00 ± 1.25 ^d^	1.13 ± 0.03 ^b^	17.49 ± 0.41 ^b^	10.51 ± 0.36 ^c^
Rosemary	25.09 ± 0.67 ^a^	17.21 ± 0.54 ^a^	98.91 ± 3.40 ^b^	37.20 ± 3.85 ^a^	1.68 ± 0.04 ^a^	26.09 ± 1.73 ^a^	21.93 ± 2.82 ^a^
Bay	18.56 ± 0.65 ^b^	1.74 ± 1.01 ^e^	58.85 ± 1.19 ^d^	18.78 ± 4.73 ^b^	0.16 ±0.04 ^d^	9.67 ± 0.69 ^c^	18.53 ± 0.16 ^ab^
Sage	21.43 ± 0.51 ^ab^	3.98 ± 0.56 ^d^	73.78 ± 1.49 ^cd^	9.52 ± 3.42 ^cd^	1.08 ± 0.04 ^b^	17.69 ± 0.13 ^b^	17.64 ± 0.08 ^ab^
Dill	7.26 ± 0.31 ^c^	3.19 ± 1.85 ^d^	13.02 ± 1.15 ^e^	7.35 ± 2.67 ^d^	0.56 ± 0.05 ^c^	13.09 ± 0.50 ^bc^	10.35 ± 0.33 ^c^
Parsley	4.02 ± 0.14 ^d^	1.85 ± 1.18 ^e^	7.16 ± 0.34 ^f^	9.48 ± 1.19 ^cd^	0.93 ± 0.06 ^bc^	7.22 ± 0.35 ^cd^	10.98 ± 0.33 ^c^
Fenugreek	4.34 ± 1.99 ^d^	1.48 ± 1.21 ^e^	3.31 ± 0.14 ^g^	4.22 ± 0.13 ^e^	0.58 ± 0.09 ^c^	3.19 ± 0.29 ^e^	7.61 ± 0.21 ^d^

Results are reported on dry weight basis in triplicate; the values with significant difference (*p* ≤ 0.05) are indicated by superscript letters (^a–h^).

**Table 3:** Characterization of polyphenolic compounds from Australian-grown herbs by LC-MS/MS-QTOF.

No	Proposed Compounds	Molecular Formula	RT (min)	Ionization ESI (+/−)	Molecular Weight	Theoretical (*m*/*z*)	Observed (*m*/*z*)	Mass Error (ppm)	MS/MS Product Ions	Herbs	
	**Phenolic acids**										
	**Hydroxybenzoic acids**										
1	Gallic acid	C_7_H_6_O_5_	10.544	[M − H]^−^	170.0215	169.0142	169.0144	0.3	125	* Bl, M	
2	3-*O*-Methylgallic acid	C_8_H_8_O_5_	10.632	[M − H]^−^	184.0372	183.0299	183.0294	−2.7	168, 140, 124	T	
3	Gallic acid 4-*O*-glucoside	C_13_H_16_O_10_	10.713	[M − H]^−^	332.0743	331.0670	331.0662	−2.4	169, 125	* T, By	
4	3,4-*O*-Dimethylgallic acid	C_9_H_10_O_5_	12.632	** [M + H]^+^	198.0528	199.0601	199.0595	3.0	153, 139, 125, 111	* Bl, M, R, S, T	
5	Protocatechuic acid 4-*O*-glucoside	C_13_H_16_O_9_	12.620	[M − H]^−^	316.0794	315.0721	315.0716	−1.6	153	* R, T, M, Bl	
6	2,3-Dihydroxybenzoic acid	C_7_H_6_O_4_	14.097	[M − H]^−^	154.0266	153.0193	153.0186	−4.6	109	* R, M, T, Bl	
7	4-Hydroxybenzoic acid 4-*O*-glucoside	C_13_H_16_O_8_	16.006	[M − H]^−^	300.0845	299.0772	299.0756	−0.7	255, 137	* Bl, T, S	
8	2-Hydroxybenzoic acid	C_7_H_6_O_3_	17.155	[M − H]^−^	138.0317	137.0244	137.0238	−0.6	93	* T, R, M, S, By, Bl, O	
**Hydroxycinnamic acids**											
9	Caffeoyl glucose	C_15_H_18_O_9_	14.513	[M − H]^−^	342.0951	341.0878	341.0877	−0.3	179, 161	* T, R, Bl	
10	Caffeoyl tartaric acid	C_13_H_12_O_9_	15.864	[M − H]^−^	312.0481	311.0408	311.0401	−2.3	161	* M, Bl	
11	3-*p*-Coumaroylquinic acid	C_16_H_18_O_8_	16.456	[M − H]^−^	338.1002	337.0929	337.0929	0.0	265, 173, 162	* M, R, S	
12	*p*-Coumaric acid 4-*O*-glucoside	C_15_H_18_O_8_	16.984	[M − H]^−^	326.1002	325.0929	325.0917	−3.7	163	* T, Bl	
13	3-Caffeoylquinic acid	C_16_H_18_O_9_	17.680	[M − H]^−^	354.0951	353.0878	353.0870	−2.3	253, 190, 144	* T, M, R, S, Bl	
14	3-Feruloylquinic acid	C_17_H_20_O_9_	17.858	[M − H]^−^	368.1107	367.1034	367.1032	−0.5	298, 288, 192, 191	M	
15	Sinapic acid	C_11_H_12_O_5_	18.318	[M + H]^+^	224.0685	225.0758	225.0760	0.9	193, 179, 149 134,	Bl, O	
16	*m*-Coumaric acid	C_9_H_8_O_3_	20.039	** [M − H]^−^	164.0473	163.0400	163.0405	3.1	119	* Bl, R, O, S, By	
17	Ferulic acid 4-*O*-glucoside	C_16_H_20_O_9_	20.816	[M − H]^−^	356.1107	355.1034	355.1028	−1.7	193, 178, 149, 134	* R, S	
18	Feruloyl tartaric acid	C_14_H_14_O_9_	21.620	[M − H]^−^	326.0638	325.0565	325.0542	−4.6	193, 149	* S, M, Bl	
19	Caffeic acid	C_9_H_8_O_4_	21.084	[M − H]^−^	180.0423	179.0350	179.0345	−1.7	161, 135	* Bl, R, M, O, S, T	
20	Ferulic acid	C_10_H_10_O_4_	21.595	[M − H]^−^	194.0579	193.0506	193.0501	−2.6	178, 149, 134	* S, Bl	
21	*p*-Coumaroyl tartaric acid	C_13_H_12_O_8_	20.039	** [M − H]^−^	296.0532	295.0459	295.0446	−1.7	115	* Bl, S	
22	Chicoric acid	C_22_H_18_O_12_	30.115	** [M − H]^−^	474.0798	473.0725	473.0736	2.3	293, 311	Bl	
23	Rosmarinic acid	C_18_H_16_O_8_	33.487	[M − H]^−^	360.0845	359.0772	359.0754	−3.2	179, 161, 135	* R, T, M, S, O, Bl, By	
24	Cinnamoyl glucose	C_15_H_18_O_7_	42.212	[M − H]^−^	310.1053	309.0980	309.0995	4.9	147, 131, 103	* T, M, O, R, S	
**Hydroxyphenyl acetic acids**											
25	3,4-Dihydroxyphenylacetic acid	C_8_H_8_O_4_	12.532	[M − H]^−^	168.0423	167.0350	167.0349	−0.6	149, 123	* R, M, T, Bl	
**Hydroxyphenylpentanoic acids**											
26	5-(3′,4′,-dihydroxyphenyl)-γ-valerolactone	C_11_H_12_O_4_	41.689	[M − H]^−^	208.0736	207.0663	207.0653	−4.8	163, 119	S	
**Hydroxyphenylpropanoic acids**											
27	3-Hydroxy-3-(3-hydroxyphenyl)propionic acid	C_9_H_10_O_4_	14.984	[M − H]^−^	182.0579	181.0506	181.0512	3.3	163, 135, 119	* T, S	
28	Dihydroferulic acid 4-*O*-glucuronide	C_16_H_20_O_10_	22.759	[M − H]^−^	372.1056	371.0983	371.1014	3.4	195	* T, R, S	
	**Flavonoids**										
	**Flavanols**										
29	(+)-Gallocatechin	C_15_H_14_O_7_	16.244	[M − H]^−^	306.0740	305.0667	305.0639	−4.3	261, 219	* By, S	
30	(+)-Catechin	C_15_H_14_O_6_	21.158	[M − H]^−^	290.0790	289.0710	289.0701	−0.3	245, 205, 179	By	
31	3′-*O*-Methyl-(−)-epicatechin-7-*O*-glucuronide	C_22_H_24_O_12_	25.668	[M − H]^−^	480.1268	479.1195	479.1184	−2.3	149, 121	R	
32	4′-*O*-Methyl-(−)-epigallocatechin-7-*O*-glucuronide	C_22_H_24_O_13_	28.364	[M − H]^−^	496.1217	495.1144	495.1155	2.2	451, 313	T	
33	Chrysoeriol 7-*O*-glucoside	C_22_H_22_O_11_	40.831	[M − H]^−^	462.1162	461.1089	461.1075	−3.0	299, 285	* R, S, By	
**Flavones**											
34	Apigenin 6,8-di-C-glucoside	C_27_H_30_O_15_	20.343	[M − H]^−^	594.1585	593.1512	593.1515	0.5	503, 473	* R, M, By	
35	Apigenin 6-C-glucoside	C_21_H_20_O_10_	27.103	[M − H]^−^	432.1056	431.0983	431.0979	−0.9	413, 341, 311	* T, R, S, By	
36	6-Hydroxyluteolin 7-*O*-rhamnoside	C_21_H_20_O_11_	28.258	** [M−H]^−^	448.1006	447.0933	447.0926	−1.6	301	* R, M, S, T, D, By, Bl, F	
37	Luteolin 7*-O*-glucuronide	C_21_H_18_O_12_	28.447	[M − H]^−^	462.0798	461.0725	461.0716	−2.4	285, 216	* M, T, R, S, O	
38	Rhoifolin	C_27_H_30_O_14_	30.045	[M − H]^−^	578.1636	577.1563	577.1525	−4.3	413, 269	* R, M, S, D, F	
39	Apigenin 7-*O*-glucuronide	C_21_H_18_O_11_	32.538	[M − H]^−^	446.0849	445.0776	445.0765	−3.5	269, 175	* T, R, S	
**Flavanones**											
40	Neoeriocitrin	C_27_H_32_O_15_	25.227	[M − H]^−^	596.1741	595.1668	595.1669	0.2	431, 287	* R, M, S	
41	Narirutin	C_27_H_32_O_14_	28.232	[M − H]^−^	580.1792	579.1719	579.1708	−1.9	271	M	
42	Hesperetin 3′-*O*-glucuronide	C_22_H_22_O_12_	29.202	[M−H]^−^	478.1111	477.1038	477.1022	−3.4	301, 175, 113, 85	* R, S	
43	Hesperidin	C_28_H_34_O_15_	30.888	[M − H]^−^	610.1898	609.1825	609.1803	−3.6	301	R	
44	Naringenin 7-*O*-glucoside	C_21_H_22_O_10_	31.079	[M − H]^−^	434.1213	433.1140	433.1136	−0.9	373, 343, 303	T	
**Flavonols**											
45	Kaempferol 3,7-*O*-diglucoside	C_27_H_30_O_16_	24.228	[M − H]^−^	610.1534	609.1461	609.1464	0.5	447, 285	* R, M, S, T, By	
46	Myricetin 3-*O*-rhamnoside	C_21_H_20_O_12_	28.520	[M − H]^−^	464.0955	463.0882	463.0887	0.7	317	* Bl, R, S, T, By	
47	Quercetin 3′-*O*-glucuronide	C_21_H_18_O_13_	25.113	[M − H]^−^	478.0747	477.0674	477.0676	0.4	301	* R, S	
48	Quercetin 3-*O*-(6″-malonyl-glucoside)	C_24_H_22_O_15_	28.196	[M − H]^−^	550.0959	549.0886	549.0886	0.0	445, 300, 160	* T, Bl	
49	Isorhamnetin 3-*O*-glucuronide	C_22_H_20_O_13_	29.864	[M − H]^−^	492.0904	491.0831	491.0822	−1.8	315, 300, 272, 255	* R, S	
50	Quercetin 3-*O*-arabinoside	C_20_H_18_O_11_	30.940	[M − H]^−^	434.0849	433.0776	433.0756	−4.6	301	By	
51	Isorhamnetin	C_16_H_12_O_7_	36.775	[M − H]^−^	316.0583	315.0510	315.0498	−3.8	300, 271	* M, S	
52	3,7-Dimethylquercetin	C_17_H_14_O_7_	45.315	** [M − H]−	330.0740	329.0667	329.0660	−1.1	314, 299, 271	* M, O, R, S, Bl	
**Dihydroflavonols**											
53	Dihydromyricetin 3-*O*-rhamnoside	C_21_H_22_O_12_	15.838	[M − H]^−^	466.1111	465.1038	465.1024	−3.0	301	T	
54	Dihydroquercetin	C_15_H_12_O_7_	28.746	[M − H]^−^	304.0583	303.0510	303.0496	−4.6	285, 275, 151	* T, O	
**Dihydrochalcones**											
55	Phloretin 2′-*O*-xylosyl-glucoside	C_26_H_32_O_14_	21.931	[M − H]^−^	568.1792	567.1719	567.1696	−4.1	437, 275, 169	* R, S	
**Anthocyanins**											
56	Cyanidin 3-*O*-(6″-p-coumaroyl-glucoside)	C_30_H_27_O_13_	20.896	[M + H]^+^	595.1452	596.1525	596.1527	0.3	287	* T, M, O	
57	Quercetin 3-*O*-xylosyl-glucuronide	C_26_H_26_O_17_	36.011	[M + H]^+^	610.1170	611.1243	611.1247	0.7	679, 303, 285, 239	* T, O	
**Isoflavonoids**											
58	Dihydrobiochanin A	C_16_H_14_O_5_	4.146	[M + H]^+^	286.0841	287.0914	287.0914	0.0	269, 203, 175	O	
59	3′,4′,5,7-Tetrahydroxyisoflavanone	C_15_H_12_O_6_	34.208	[M − H]^−^	288.0634	287.0561	287.0564	1.0	269, 259	* T, O, Bl	
60	5,6,7,3′,4′-Pentahydroxyisoflavone	C_15_H_10_O_7_	40.677	[M − H]^−^	302.0427	301.0354	301.0346	−2.7	274, 200, 136	* T, O, S, By	
61	3′,4′,7-Trihydroxyisoflavanone	C_15_H_12_O_5_	45.000	[M − H]^−^	272.0685	271.0612	271.0609	−1.1	177, 151, 119, 107	* M, T, O	
62	Sativanone	C_17_H_16_O_5_	46.952	[M − H]^−^	300.0998	299.0925	299.0921	−1.3	284, 269, 225	S	
63	3′-Hydroxydaidzein	C_15_H_10_O_5_	47.113	[M − H]^−^	270.0528	269.0455	269.0451	−1.5	241, 224, 213, 181	* R, T, M, Bl, O, S	
64	4′-Methoxy-2′,3,7-trihydroxyisoflavanone	C_16_H_14_O_6_	47.278	[M − H]^−^	302.0790	301.0717	301.0704	−4.3	283, 177	R	
65	3′-Hydroxymelanettin	C_16_H_12_O_6_	54.909	[M − H]^−^	300.0634	299.0561	299.0559	−0.7	284	* T, M, O, R, S	
	**Other polyphenols**										
	**Hydroxycoumarins**										
66	Esculin	C_15_H_16_O_9_	15.852	[M − H]^−^	340.0794	339.0721	339.0690	−2.9	177	* S, R	
67	Coumarin	C_9_H_6_O_2_	17.634	[M + H]^+^	146.0368	147.0441	147.0428	−4.8	103, 91	O	
68	Esculetin	C_9_H_6_O_4_	20.473	[M − H]^−^	178.0266	177.0193	177.0196	0.7	149, 133, 105, 89	* Bl, R, S, T	
69	Umbelliferone	C_9_H_6_O_3_	46.399	** [M − H]^−^	162.0317	161.0244	161.0242	−1.2	133, 117, 105	* Bl, M, O, R, S	
**Hydroxybenzoketones**											
70	2-Hydroxy-4-methoxyacetophenone 5-sulfate	C_9_H_10_O_7_S	12.281	[M − H]^−^	262.0147	261.0074	261.0076	0.8	181, 97	* R, T	
**Tyrosols**											
71	Oleoside 11-methylester	C_17_H_24_O_11_	14.451	[M − H]^−^	404.1319	403.1246	403.1238	−2.0	223, 165	R, S	
72	3,4-DHPEA-AC	C_10_H_12_O_4_	85.689	[M − H]^−^	196.0736	195.0663	195.0656	−3.6	135	O	
**Phenolic terpenes**											
73	Rosmanol	C_20_H_26_O_5_	53.370	[M − H]^−^	346.1780	345.1707	345.1693	−4.1	301	* R, S	
74	Carnosol	C_20_H_26_O_4_	79.943	[M − H]^−^	330.1831	329.1758	329.1742	−4.9	285	* R, T, S	
75	Carnosic acid	C_20_H_28_O_4_	85.366	[M − H]^−^	332.1988	331.1915	331.1907	−2.4	287	* R, S, Bl	
**Alkylphenols**											
76	3-Methylcatechol	C_7_H_8_O_2_	14.350	[M − H]−	124.0524	123.0455	123.0455	1.8	281, 187, 165	* M, T	
**Other Polyphenols**											
77	Salvianolic acid B	C_36_H_30_O_16_	32.130	[M − H]^−^	718.1534	717.1461	717.1442	−2.8	519, 339, 321, 295	* S, Bl	
78	Lithospermic acid	C_27_H_22_O_12_	35.122	[M−H]^−^	538.1111	537.1038	537.1013	−4.7	493, 339, 295	* T, O	
**Lignans**											
79	Enterolactone	C_18_H_18_O_4_	4.786	[M + H]^+^	298.1205	299.1278	299.1268	−3.3	281, 187, 165	O	
80	Sesamin	C_20_H_18_O_6_	18.227	[M − H]^−^	354.1103	353.1030	353.1021	−2.5	338, 163	P	
81	7-Oxomatairesinol	C_20_H_20_O_7_	18.661	[M + H]^+^	372.1209	373.1282	373.1286	1.1	358, 343, 328, 325	D	
82	Secoisolariciresinol	C_20_H_26_O_6_	51.345	[M − H]^−^	362.1729	361.1656	361.1652	−1.1	165, 121	S	
83	Deoxyschisandrin	C_24_H_32_O_6_	84.114	[M − H]^−^	416.2199	415.2126	415.2107	−4.6	402, 347, 361, 301	S	
**Stilbenes**											
84	Piceatannol	C_14_H_12_O_4_	8.718	[M − H]^−^	244.0736	243.0663	243.0662	−0.4	225, 201, 175, 159	* F, D	

** = compound was identified in positive and negative mode [M + H]^+^/[M − H]^−^ modes. * = proposed compound was identified in more than one sample. RT stands for “retention time”. Herbs were presented with abbreviations; Oregano (O), Rosemary (R), Basil (Bl), Bay (By), Parsley (P), Mint (M), Fenugreek (F), Dill (D), Sage (S) and Thyme (T).

**Table 4:** Pearson’s correlation between polyphenolic contents in herbs and their different anti-oxidant activities.

Variables	TPC	TFC	TTC	DPPH	FRAP	ABTS	PMAP	RPA	FICC	•OH−RSA	Phenolic Acids	Flavonoids	Phenolic Terpenes
TFC	0.62												
TTC	0.44	0.27											
DPPH	0.72	0.77 **	0.35										
FRAP	0.58	0.78 **	0.09	0.67									
ABTS	0.87 **	0.78 **	0.455	0.96 **	0.70								
PMAP	0.09	0.49	0.08	0.54	0.46	0.41							
RPA	0.28	0.53	−0.06	0.57	0.78 **	0.51	0.76 **						
FICC	0.23	0.73 *	0.07	0.46	0.64	0.48	0.20	0.37					
•OH−RSA	0.54	0.89 **	0.21	0.83 **	0.86 **	0.79 **	0.54	0.66	0.70				
Phenolic acids	0.45	0.60	−0.19	0.52	0.90 **	0.53	0.53	0.88	0.41	0.66			
Flavonoids	0.39	0.46	−0.17	0.62	0.66	0.58	0.51	0.88	0.32	0.54	0.80 **		
Phenolic terpenes	0.07	0.59	−0.18	0.39	0.80 **	0.33	0.66	0.89	0.59	0.64	0.85 **	0.71	
Stilbenoids	0.07	0.59	−0.18	0.39	0.80 **	0.33	0.66	0.89	0.59	0.64	0.85 **	0.71	1.00 **

* = Significant correlation at *p* ≤ 0.05; ** = Significant correlation at *p* ≤ 0.01.

## Data Availability

The data is available in the Supplementary Material.

## Supplementary Materials

The following are available online at https://www.mdpi.com/article/10.3390/antiox10111770/s1, Figure S1: Base Peak Chromatograms (BPC) of 10 selected herbs in negative (red) and positive mode (black), Figure S2: Chromatogram and spectrum of some selected compounds with their observed mass and retention time. Table S1: HPLC-MS semi quantification of abundant phenolic compounds in 10 herbs. Table S2: Pearson’s pairwise correlation of phenolics and antioxidant activities.
