# Supplementary material for: LC-MS/MS-QTOF Screening and Identification of Phenolic Compounds from Australian Grown Herbs and Their Antioxidant Potential
Source: Antioxidants (Basel). 2021 Nov 5;10(11):1770. doi: 10.3390/antiox10111770 (PMC8615083; doi:10.3390/antiox10111770)
Supplement: Supplementary file 1 [file antioxidants-10-01770-s001.zip › antioxidants-1417389-supplementary.pdf]

Supplementary Material

Antioxidant activities

Determination of total polyphenols (TP)

To start, 25 µL (25% Folin–Ciocalteu reagent v/v) with 200 µL water (Milli-Q) were added to 25 µL of sample extracts in 96-well plates. Then, the plate was incubated for 5 min at 25 ◦C. Finally, 25 µL (10% v/v sodium carbonate) were added in reaction mixture and placed in the dark for 60 min at 25 ◦C and absorbance was recorded at 765 nm. The TPC was quantified by constructing the standard curve against gallic acid ranging from 0 to 200 µg/mL in ethanol. The results were documented as milligram gallic acid equivalents (GAE) per gram dry weight of samples.

Determination of total flavonoids (TF)

In this, 80 µL of the sample extract were mixed with 80 µL 2% aluminum chloride solution and 120 µL sodium acetate aqueous solution (50 g/L) in 96-well plates. The reaction mixture was placed in the dark at 25 ◦C for 2.5 h and absorbance was recorded at 440 nm. Measurement of all samples were made in triplicate and TFC were quantified by constructing a standard curve against 0–50 µg/mL quercetin in methanol. The results were expressed as milligram quercetin equivalents (QE) per gram dry weight of the samples (r 2 = 0.999).

Determination of total tannins (TT)

To do this, 25 µL of sample solution was mixed with 150 µL of 4% vanillin solution in a 96-well plate. A total of 25 µL of 32% sulfuric acid was added to the mixture and allowed to incubate at 25 ◦C for 15 min and absorbance was recorded at 500 nm. Measurements for all samples was made in triplicate and quantification was done by constructing a standard curve with 0–1000 µg/mL catechin solution in methanol. The results were expressed as mg catechin equivalents (CE) per gram dry weight of the samples.

DPPH assay

To do this, 25 µL sample extract and 275 µL 0.1 M solution of DPPH in methanol were mixed in 96-well plate method. The reaction mixture was placed in the dark for 30 min at room temperature and absorbance was recorded at 517 nm. Anti-radical capacity of all the samples was estimated by constructing the standard curve against 0–50 µg/mL ascorbic acid in water. The results were expressed as milligram ascorbic acid equivalents per gram dry weight of the samples (mg AAE/g).

FRAP assay

An aliquot of 20 µL was mixed with 280 µL FRAP reagent (mixture of 20 mM ferric chloride, 10 mM TPTZ solution and 300 mM sodium acetate buffer in the *v/v* ratio of 1:1:1). The mixture was kept for 10 minutes at 37 °C. before the plate reading at 593 nm. The ferric reducing antioxidant property was subsequently calculated and expressed as mg AAE/g.

ABTS radical scavenging assay

To do this, 7 mM ABTS solution was mixed with 140 mM potassium persulfate solution. The reaction mixture was allowed to incubate in the dark for 16 h to generate an ABTS+ solution. The ABTS+ solution was diluted with ethanol to make its absorbance to 0.70 ± 0.02 at 734 nm. After this, 10 µL of sample extract was mixed with 290 µL of ABTS+ solution in 96-well plate and allowed to incubate at 25 ◦C for 6 min and the absorbance was recorded at 734 nm. The quantification was completed by constructing the standard curve against 0–150 µg/mL of ascorbic acid in water. The results were expressed as mg AAE/g.

Reducing Power Assay (RPA)

A total of 10 µL extract, 25 µL of 0.2 M phosphate buffer (pH 6.6), and 25 µL of K3[Fe(CN)6] were added, sequentially followed by incubation at 25 ◦C for 20 min. Then, 25 µL of 10% TCA solution was added to stop the reaction followed by the addition of 85 µL of water and 8.5 µL of FeCl3 and incubated for further

15 min at 25 ◦C. Next, the absorbance was measured at a wavelength of 750 nm. Ascorbic acid from 0 to 300 µg/mL was used to obtain a standard curve and data was presented in mg AAE/g.

Hydroxyl radical scavenging activity (•OH-RSA)

A 50 µL extract was mixed with 50 µL of 6 mM $FeSO_4 \cdot 7H_2O$ and 50 µL of 6 mM H_2O_2 (30%), followed by incubation at 25 ◦C for 10 min. After incubation, 50 µL of 6 mM 3-hydrooxybenzoic acid were added and absorbance was measured at a wavelength of 510 nm. Ascorbic acid from 0 to 300 µg/mL was used to obtain a standard curve and data was presented in mg AAE/g. The Fe^{2+} chelating activity of the samples were measured, according to Dinis, et al. [18] with modifications. A total of 15 µL extract was mixed with 85 µL of water, 50 µL of 2 mM ferrous chloride (with additional 1:15 dilution in water) and 50 µL of 5 mM ferrozine (with additional 1:6 dilution in water), followed by incubation at 25 ◦C for 10 min. Then the absorbance was measured at a wavelength of 562 nm. EDTA from concentrations of 0 to 50 µg/mL was used to obtain a standard curve and data was presented as mg EDTA/g.

Ferrous ion chelating activity (FICC)

A total of 15 µL extract was mixed with 85 µL of water, 50 µL of 2 mM ferrous chloride (with additional 1:15 dilution in water) and 50 µL of 5 mM ferrozine (with additional 1:6 dilution in water), followed by incubation at 25 ◦C for 10 min. Then the absorbance was measured at a wavelength of 562 nm. EDTA from concentrations of 0 to 50 µg/mL was used to obtain a standard curve and data was presented as mg EDTA/g.

Phosphomolybdate antioxidant power assay (PMAP)

For the PMA, 40 µL of each spice extract was added to 260 µL of phosphomolybdate reagent (0.6 M H_2SO_4, 0.028 M sodium phosphate and 0.004 M ammonium molybdate). The mixture was incubated at 95 ◦C for 90 min, cooled at room temperature and absorbance was measured at 695 nm. A standard curve was generated using concentrations of 0–200 µg/mL ascorbic acid and the results were expressed as mg AAE/g

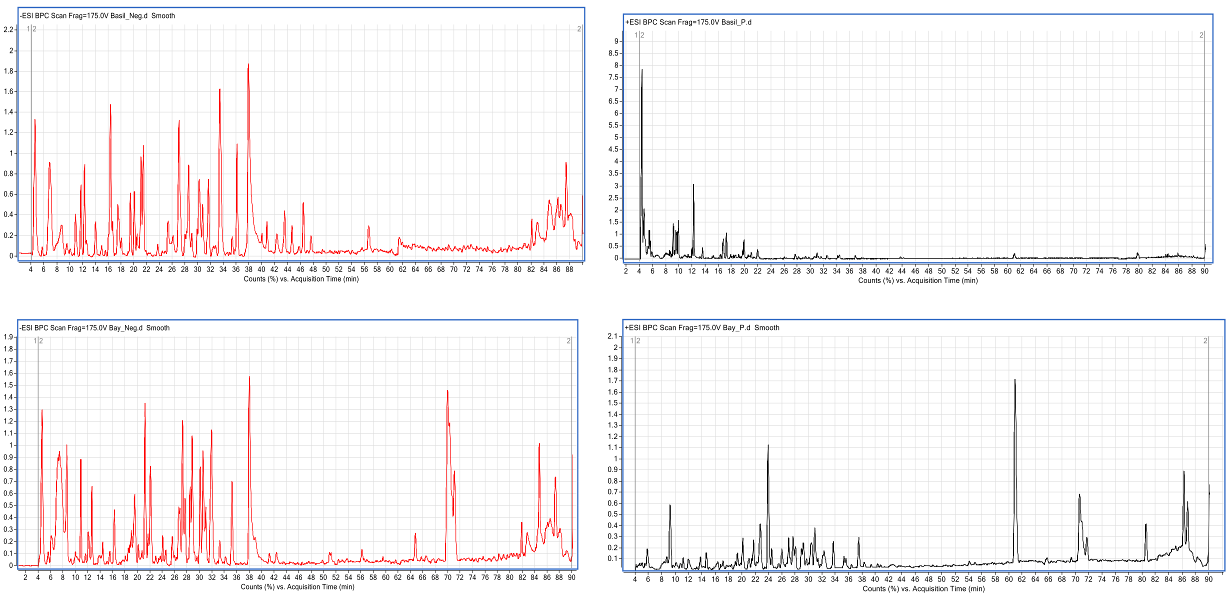

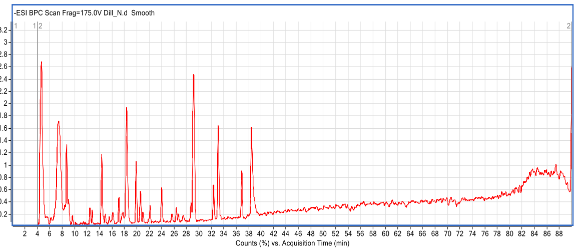
-ESI BPC Scan Frag=175.0V Dill_N.d Smooth
Counts (%) vs. Acquisition Time (min)

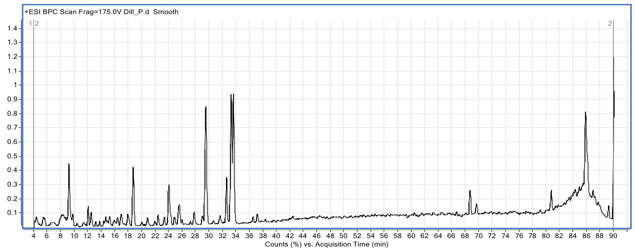
+ESI BPC Scan Frag=175.0V Dill_P.d Smooth
Counts (%) vs. Acquisition Time (min)

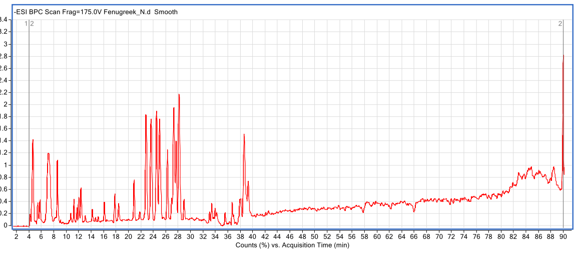
-ESI BPC Scan Frag=175.0V Fenugreek_N.d Smooth
Counts (%) vs. Acquisition Time (min)

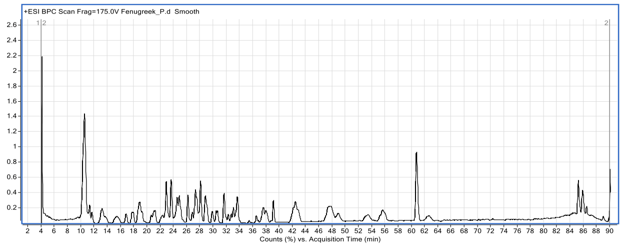
+ESI BPC Scan Frag=175.0V Fenugreek_P.d Smooth
Counts (%) vs. Acquisition Time (min)

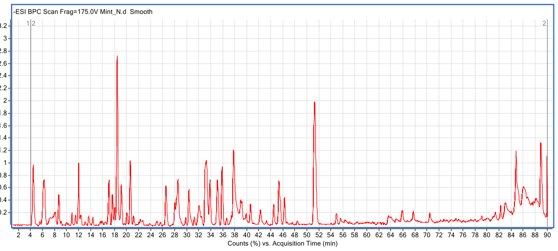
-ESI BPC Scan Frag=175.0V Mint_N.d Smooth
Counts (%) vs. Acquisition Time (min)

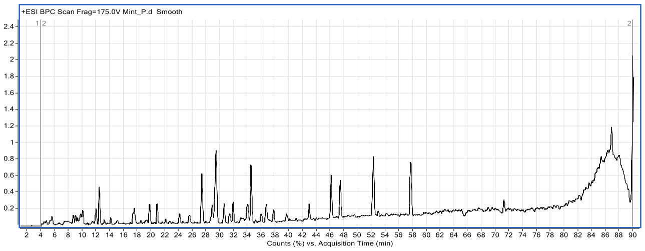
+ESI BPC Scan Frag=175.0V Mint_P.d Smooth
Counts (%) vs. Acquisition Time (min)

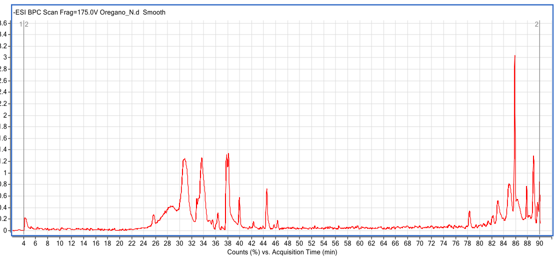
-ESI BPC Scan Frag=175.0V Oregano_N.d Smooth
Counts (%) vs. Acquisition Time (min)

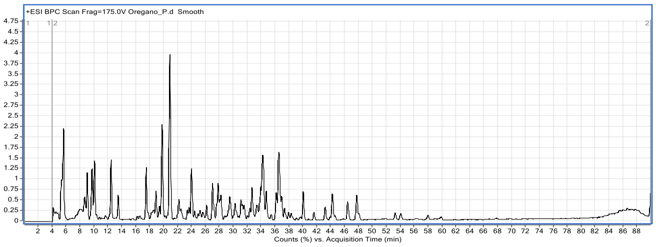
+ESI BPC Scan Frag=175.0V Oregano_P.d Smooth
Counts (%) vs. Acquisition Time (min)

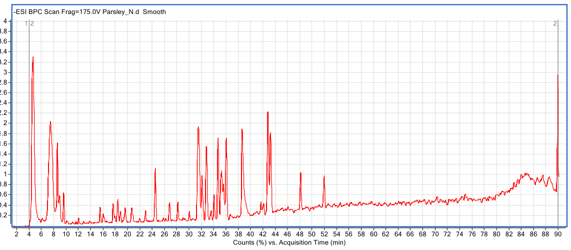
-ESI BPC Scan Frag=175.0V Parsley_N.d Smooth
Counts (%) vs. Acquisition Time (min)

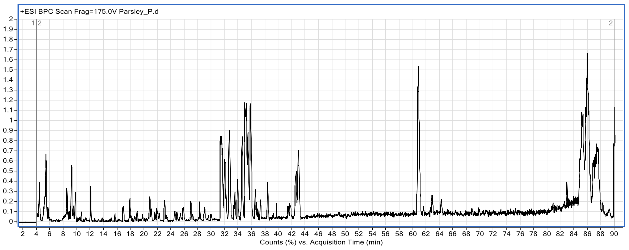
+ESI BPC Scan Frag=175.0V Parsley_P.d
Counts (%) vs. Acquisition Time (min)

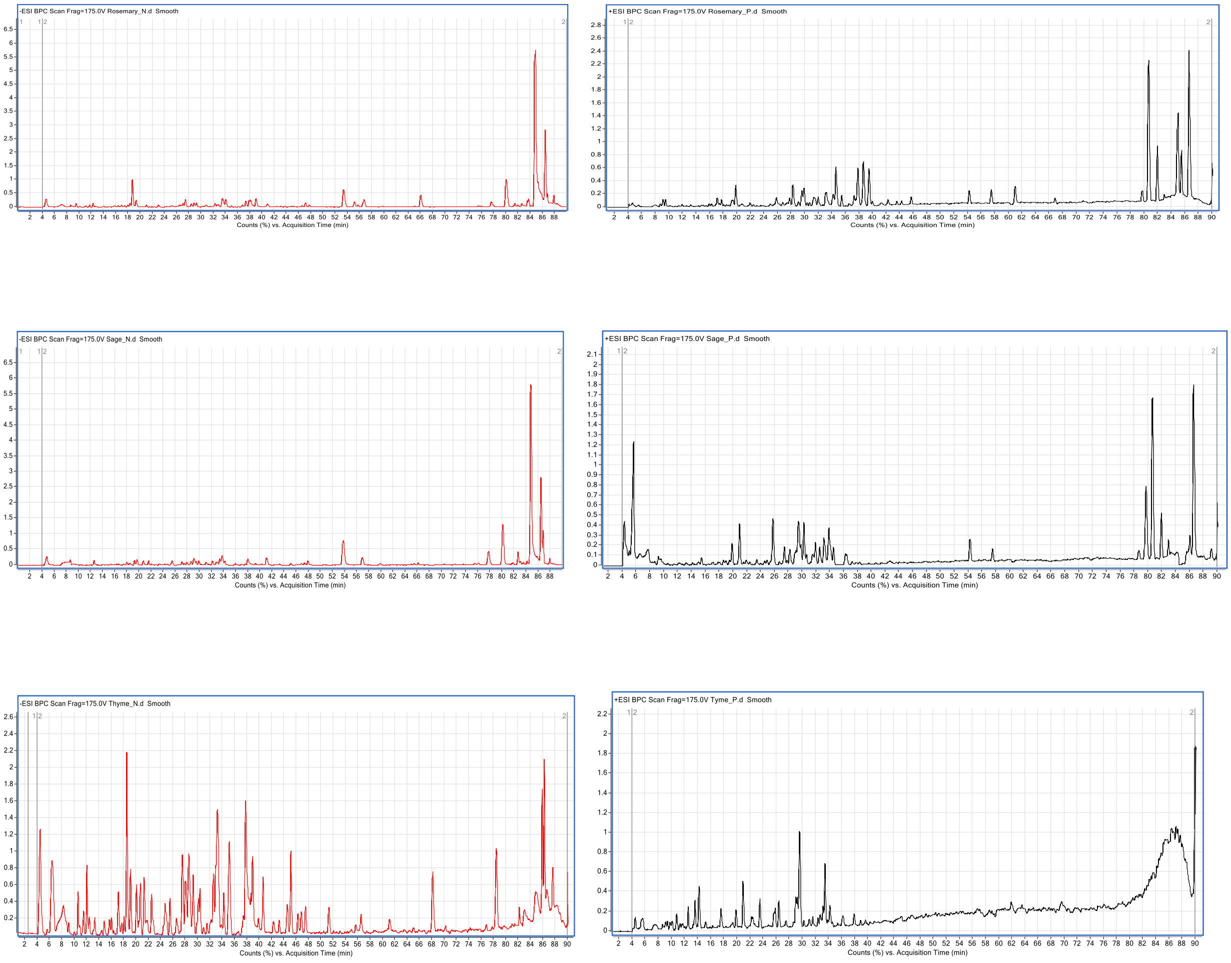

Figure S1. Base Peak Chromatograms (BPC) of 10 herbs in negative (red)and positive mode (black).

Compound 1

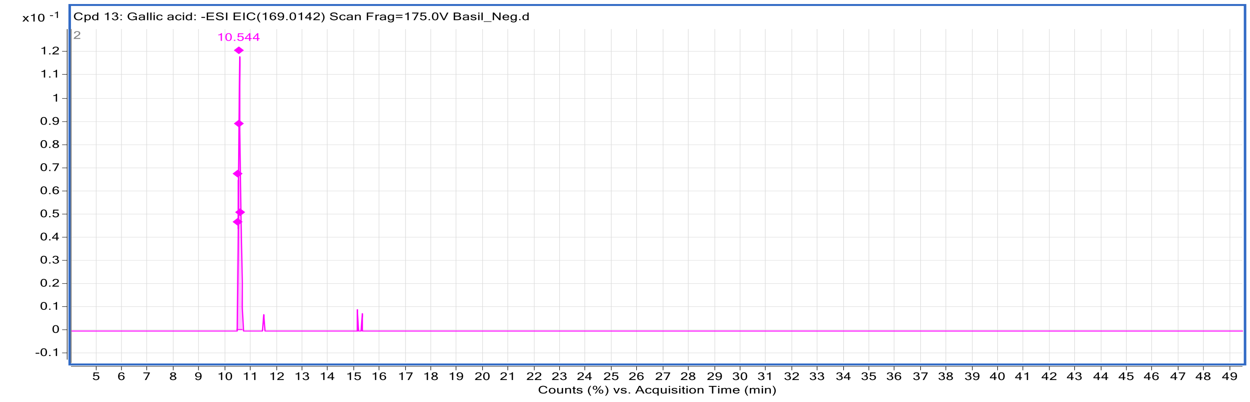

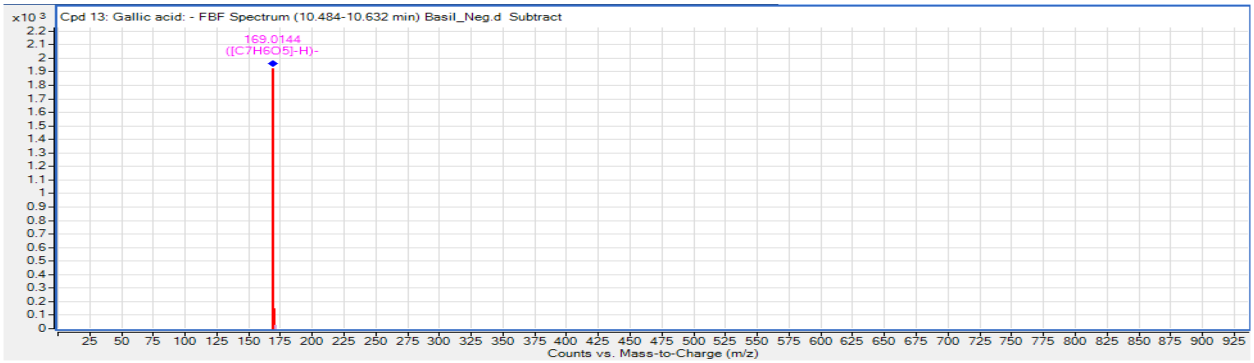

Compound 2

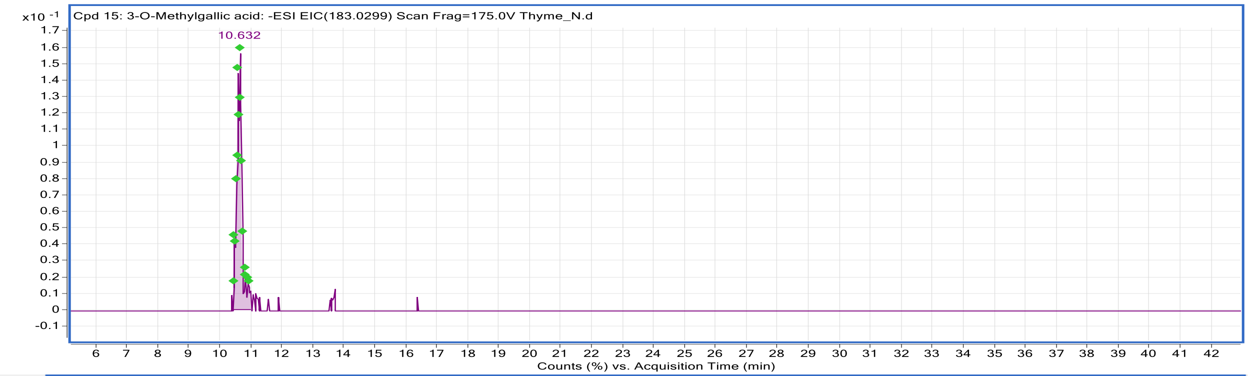

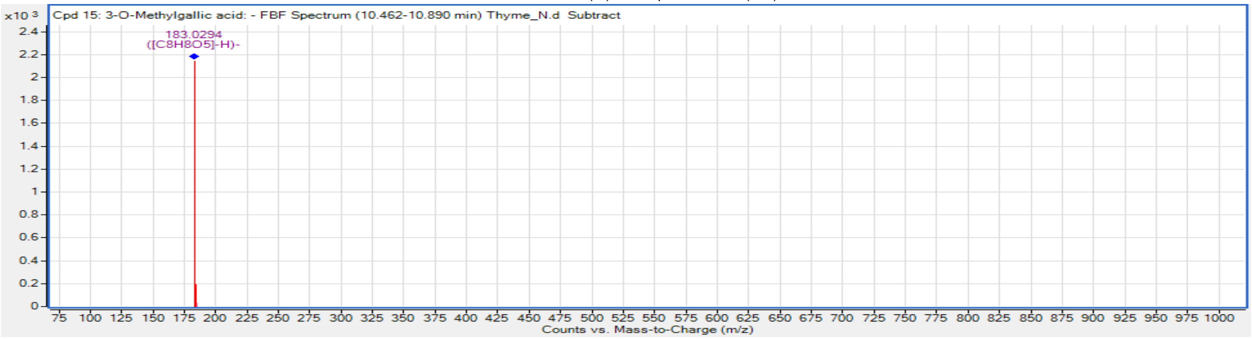

Compound 7

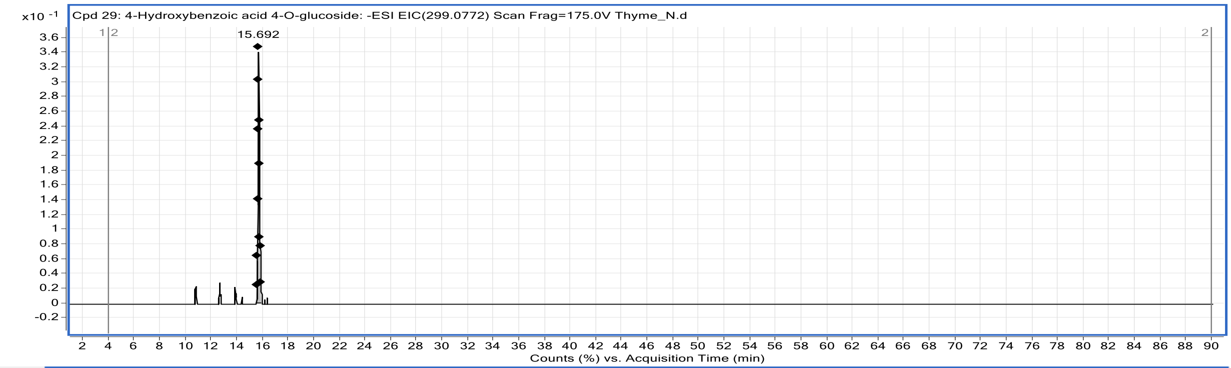

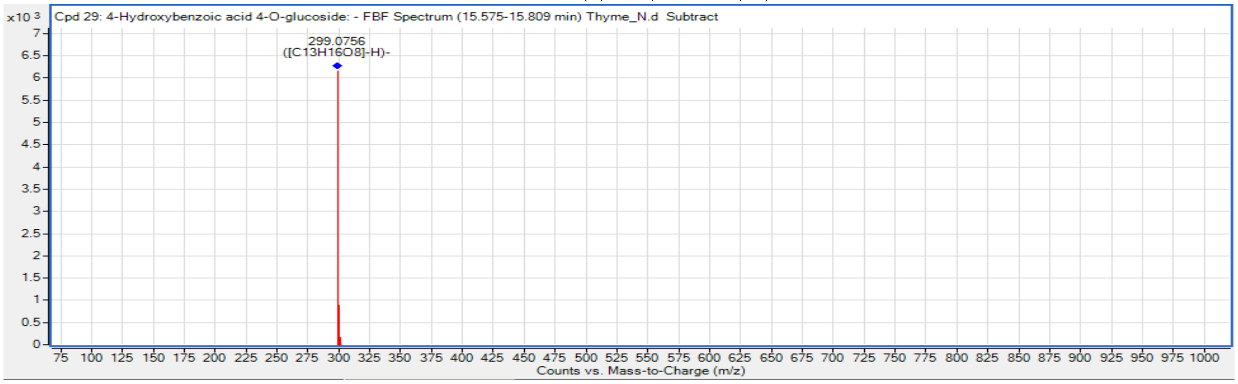

Compound 16

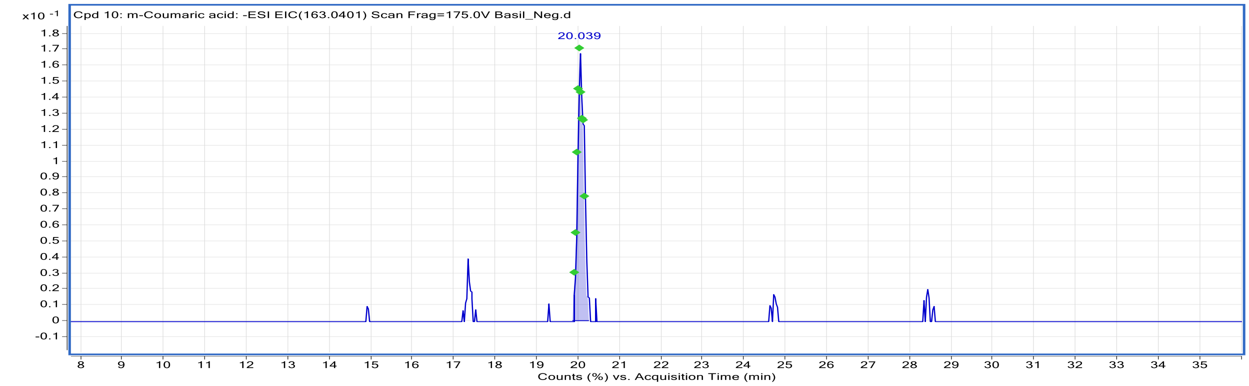

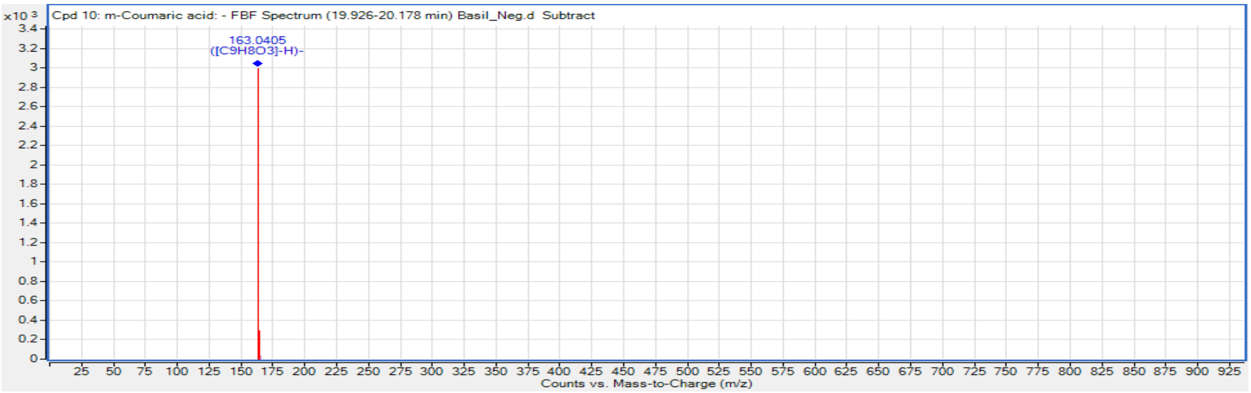

Compound 18

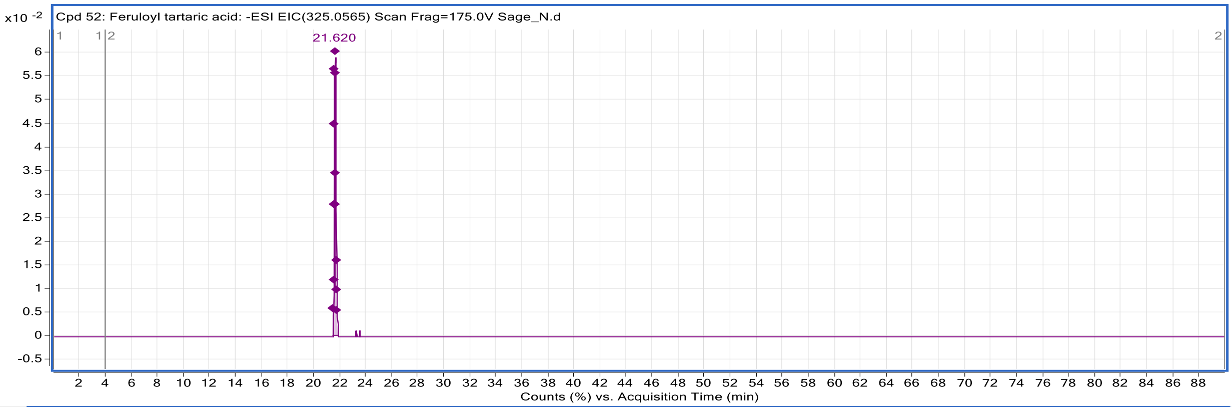

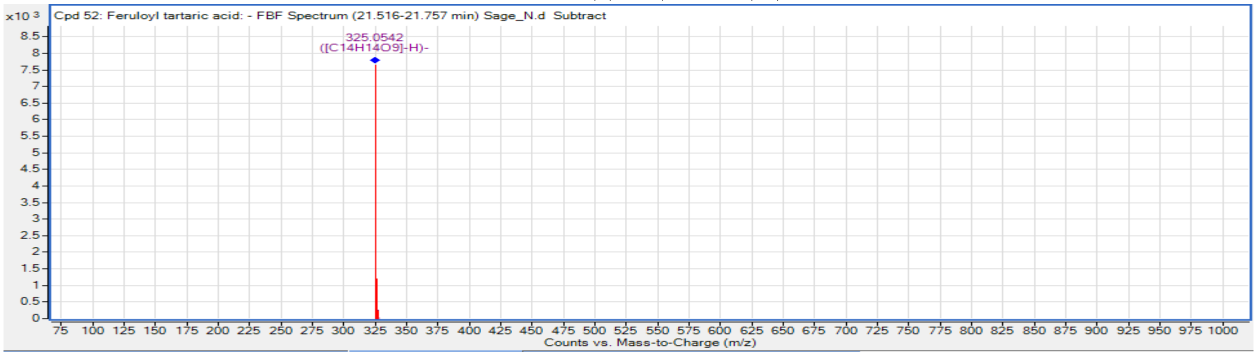

Compound 19

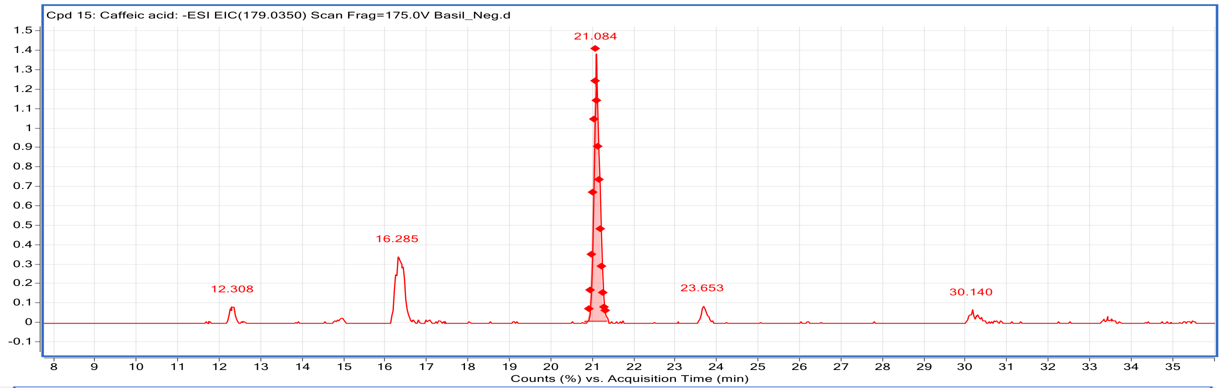

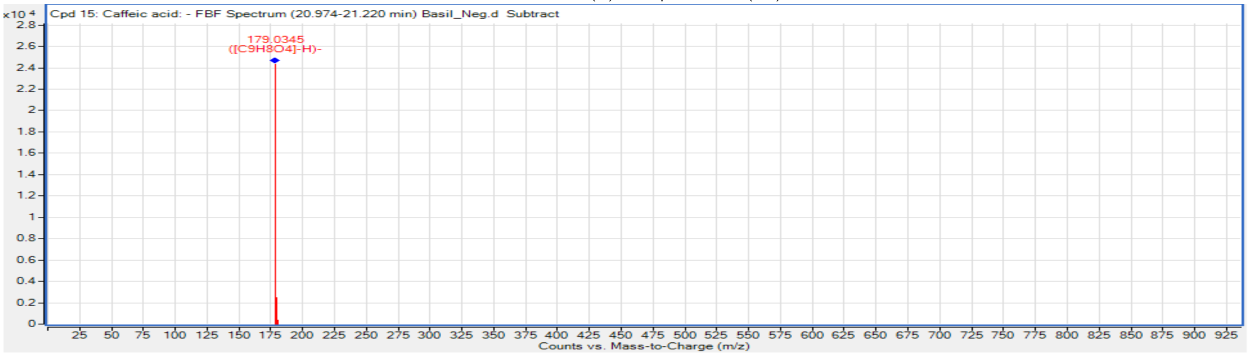

Compound 21

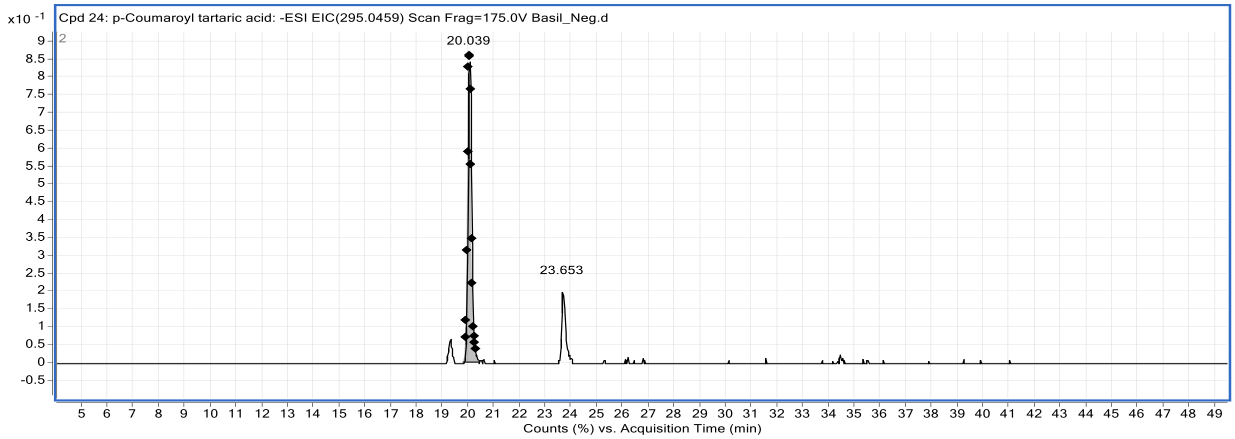

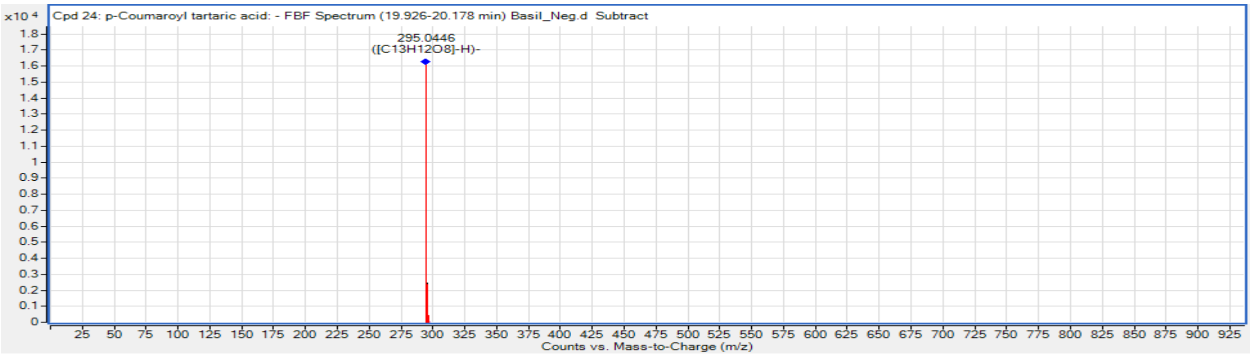

Compound 22

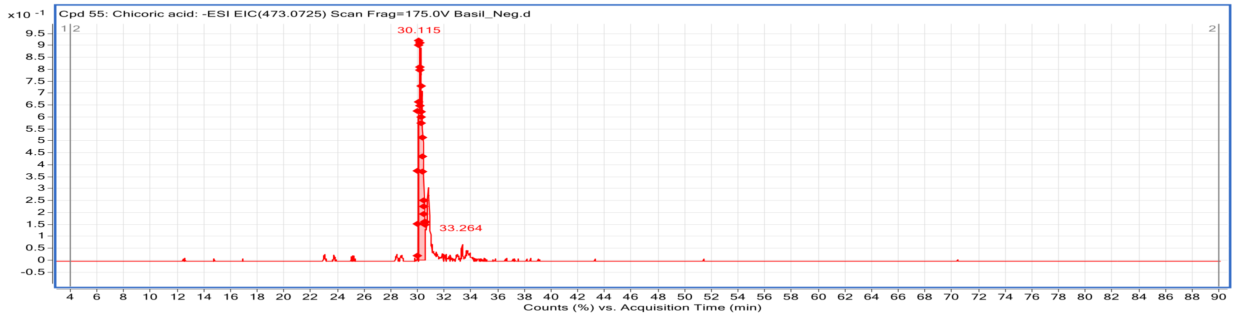

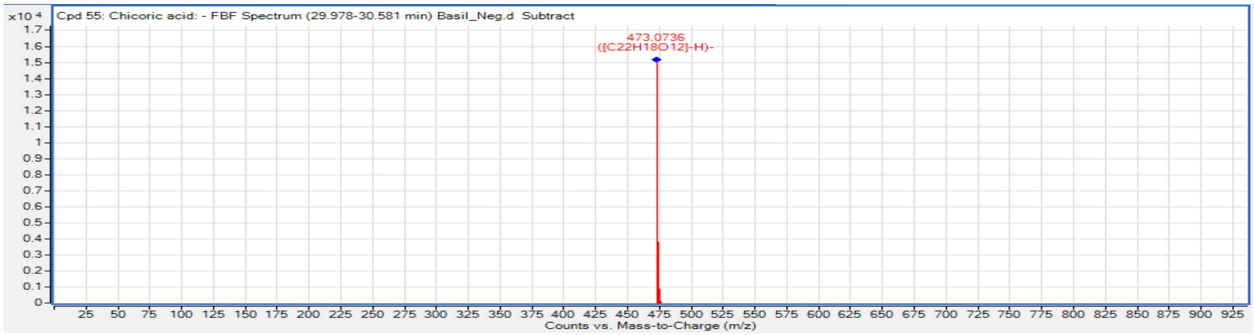

Compound 23

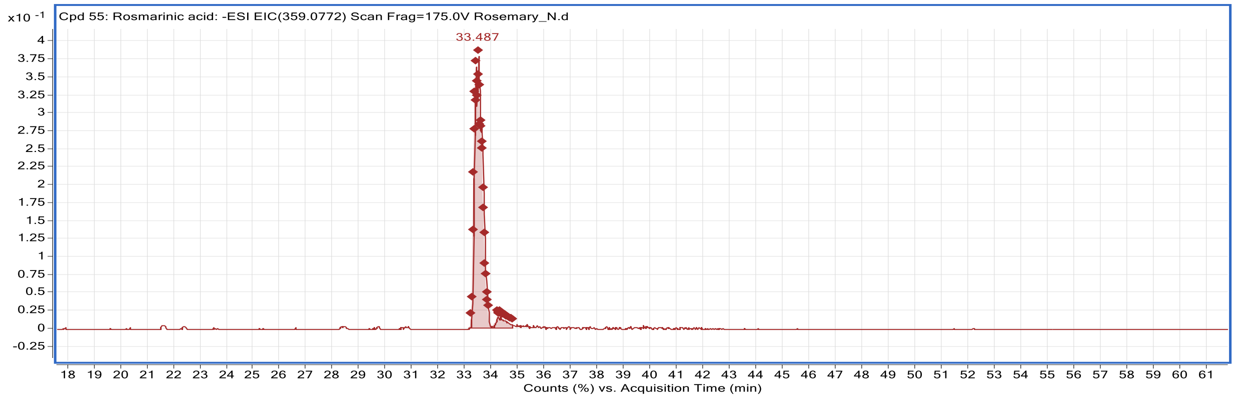

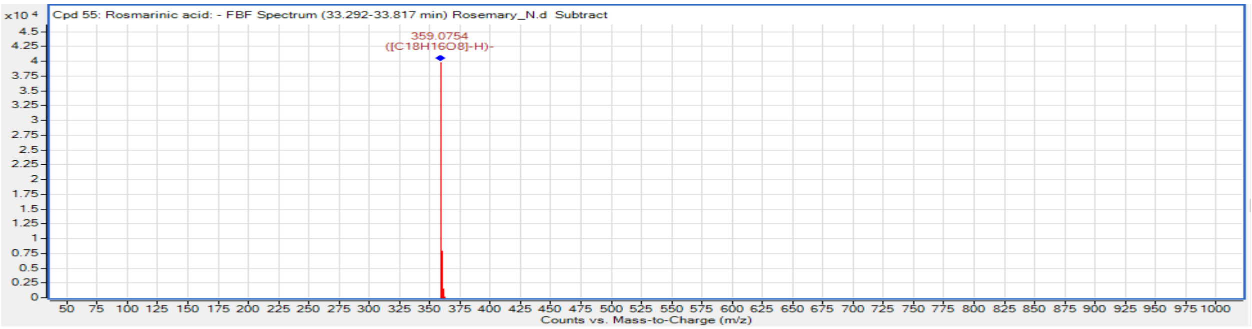

Compound 28

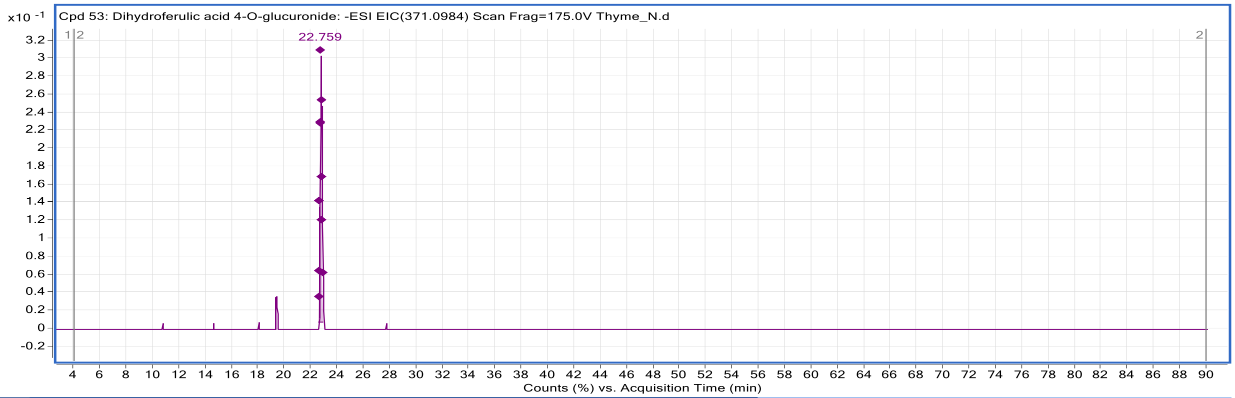

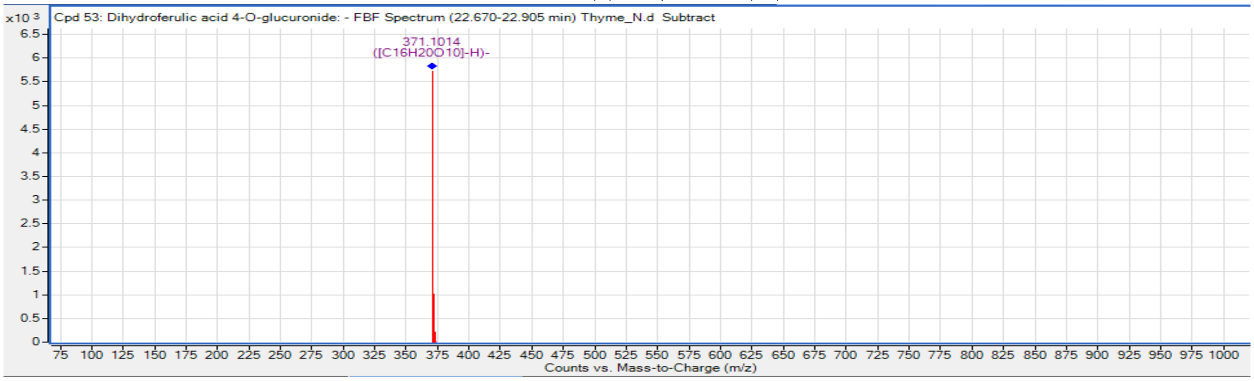

Compound 29

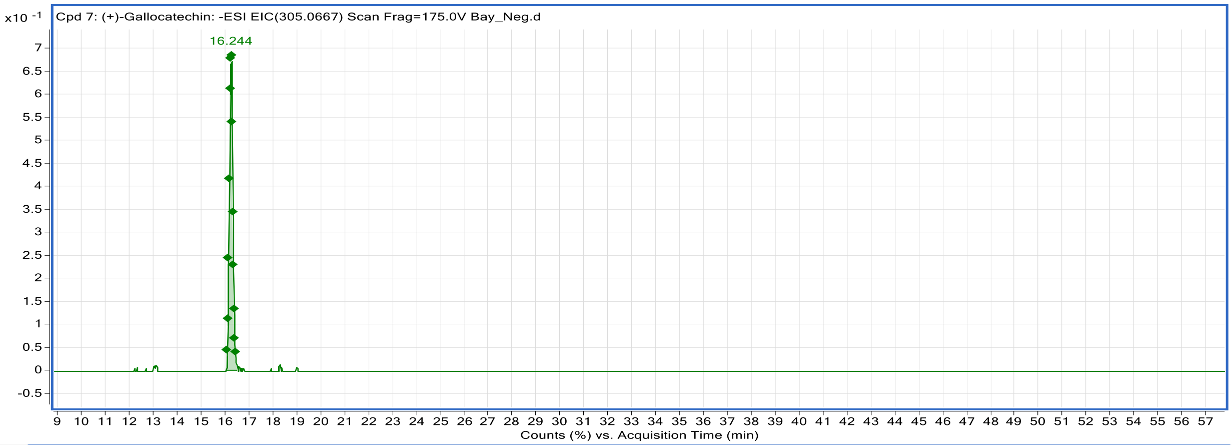

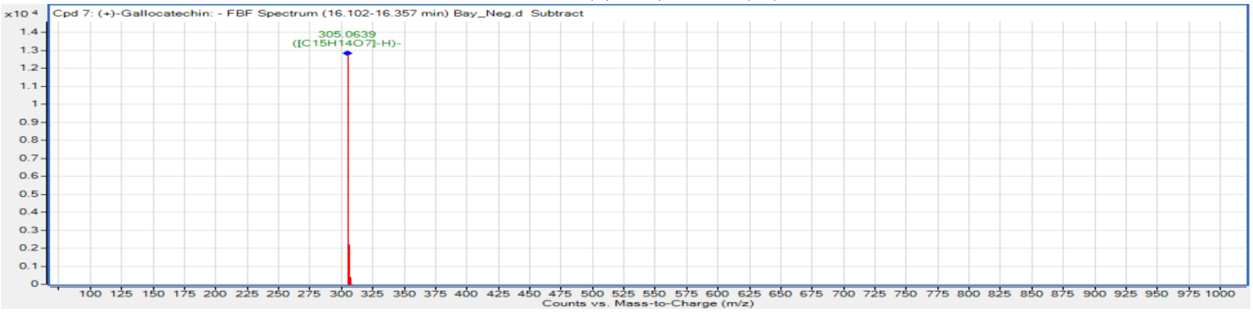

Compound 30

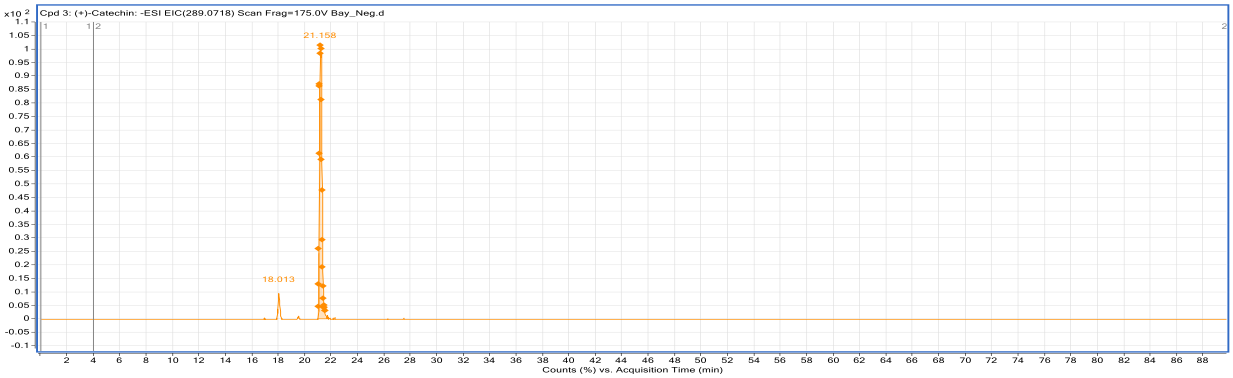

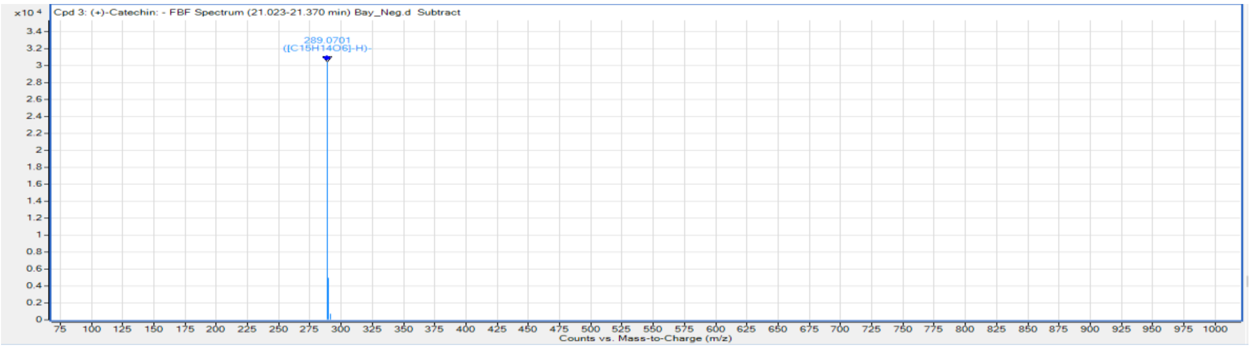

Compound 38

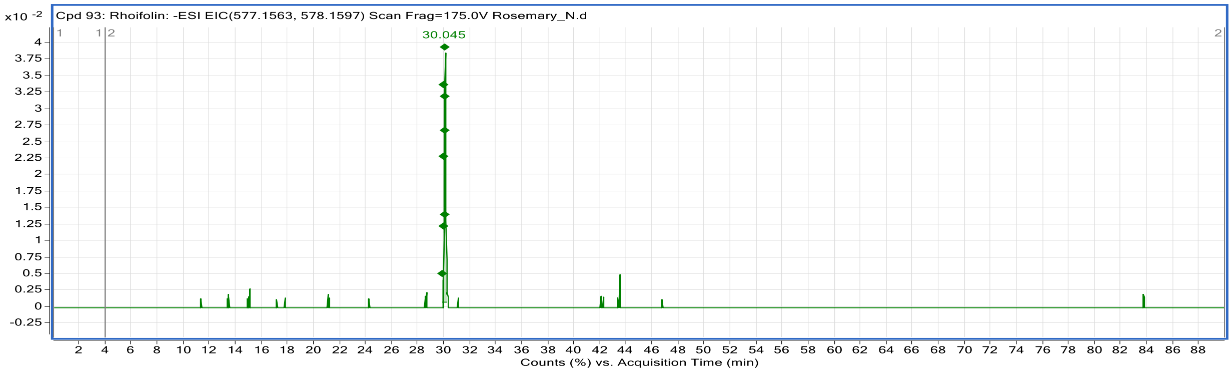

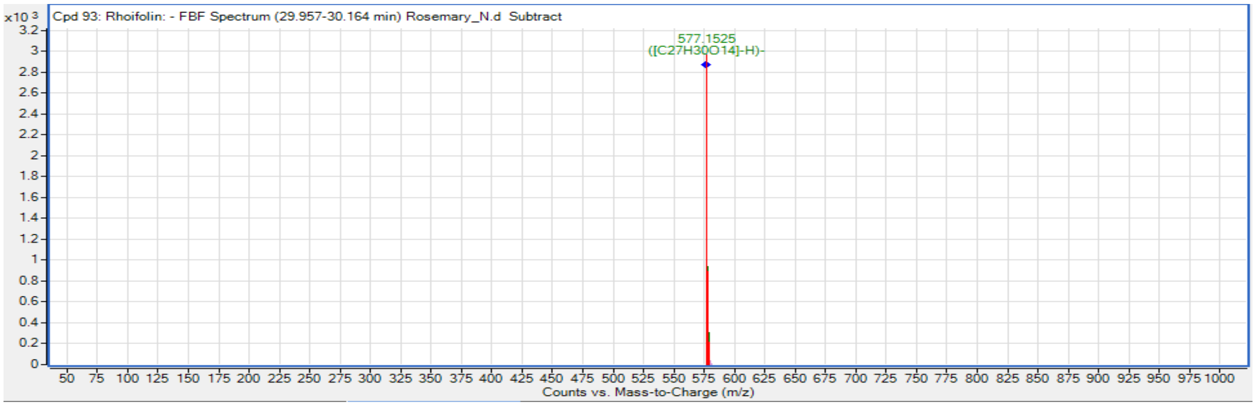

Compound 39

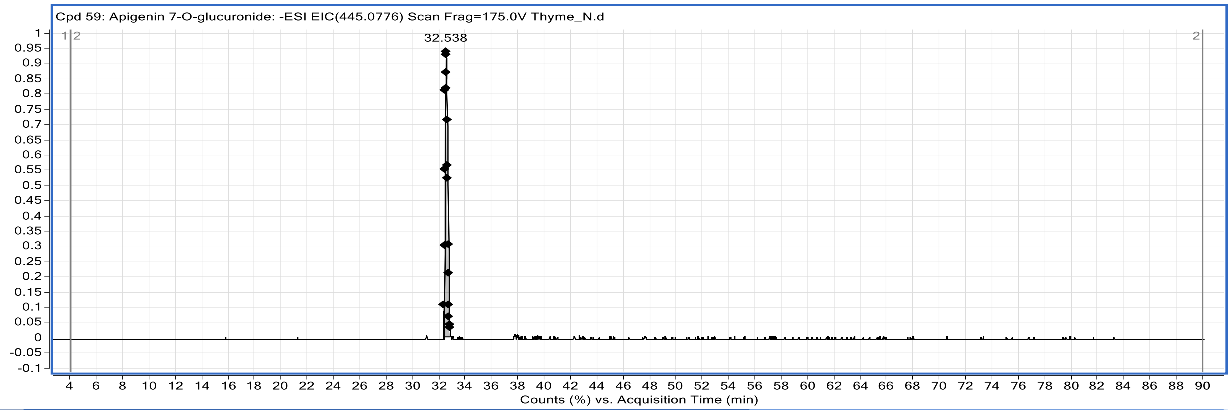

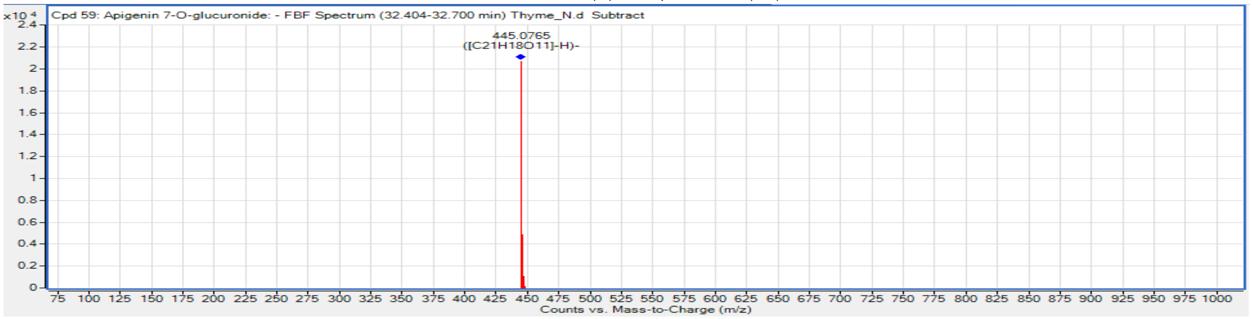

Compound 43

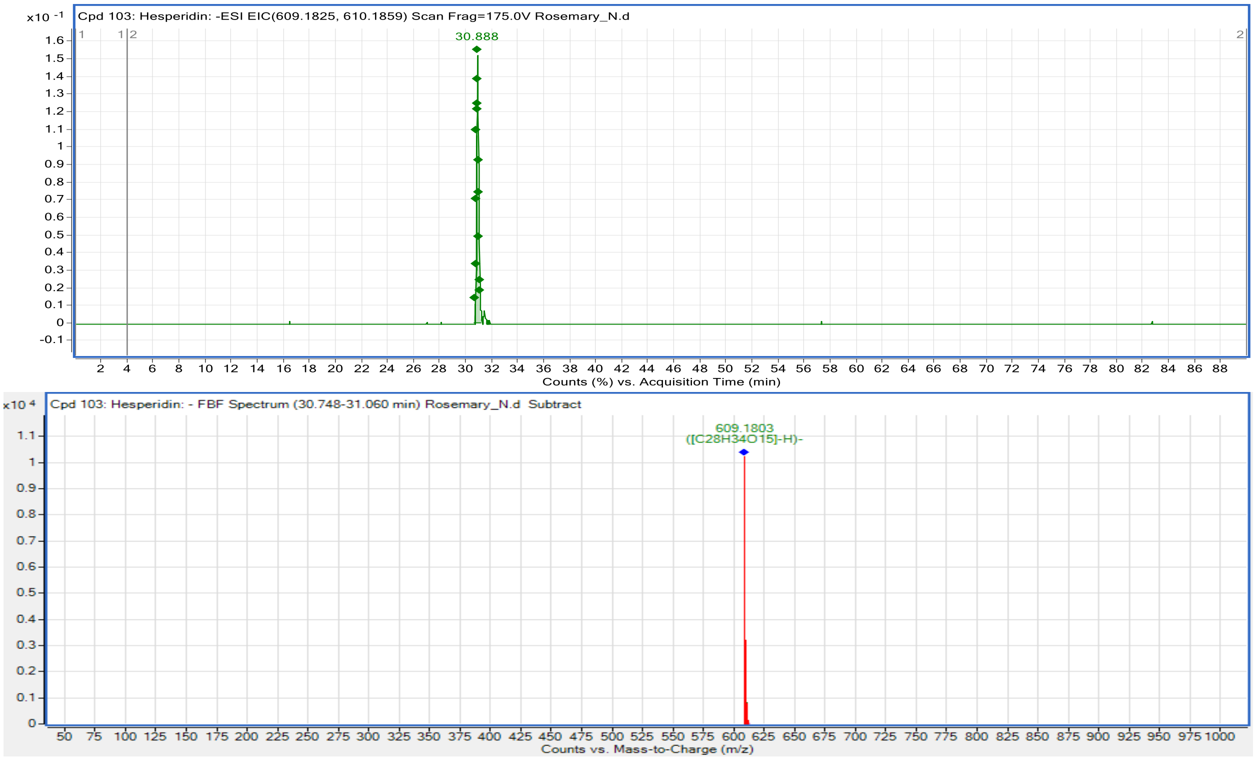

Compound 46

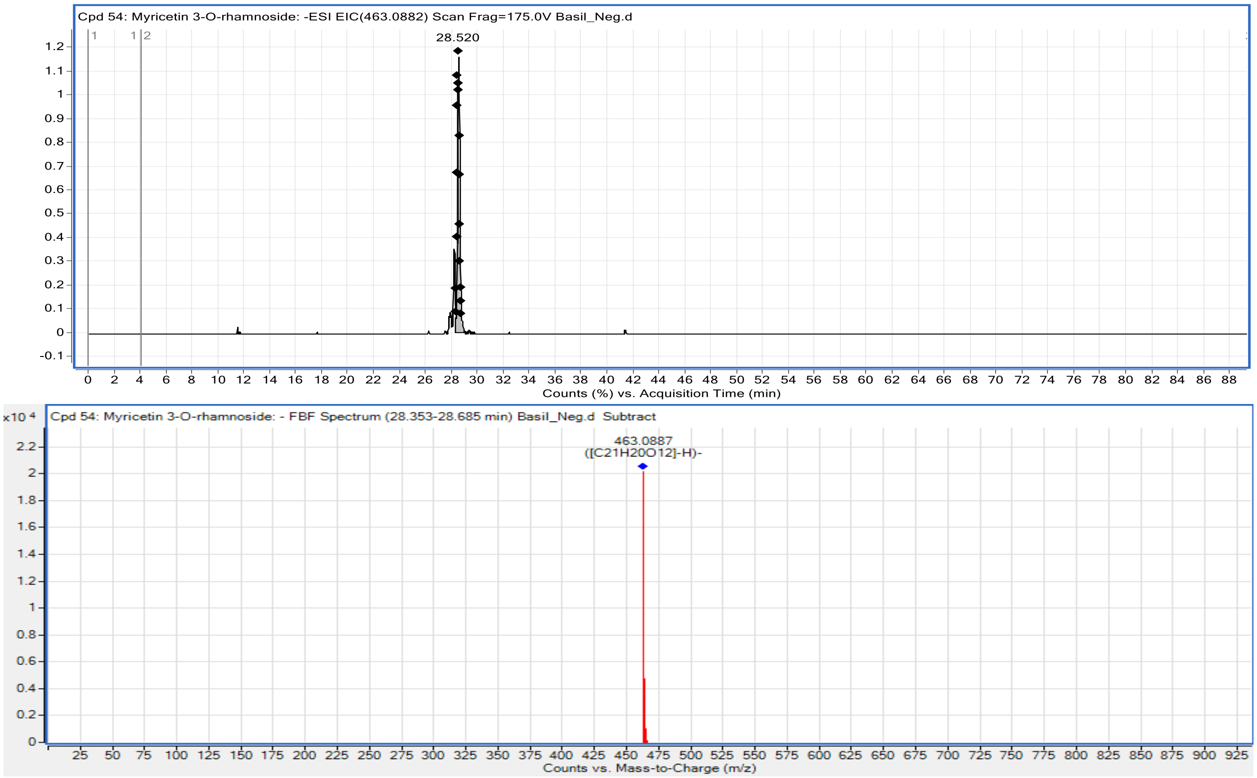

Compound 52

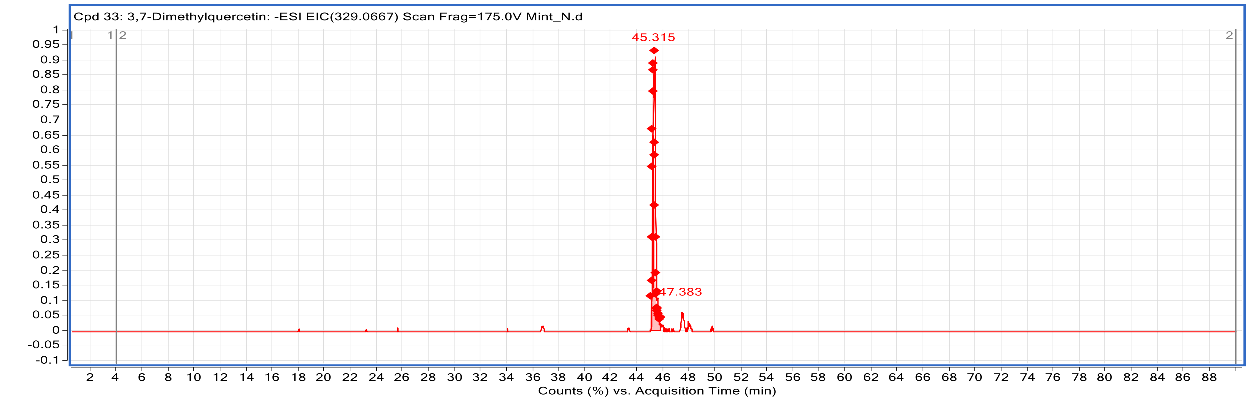

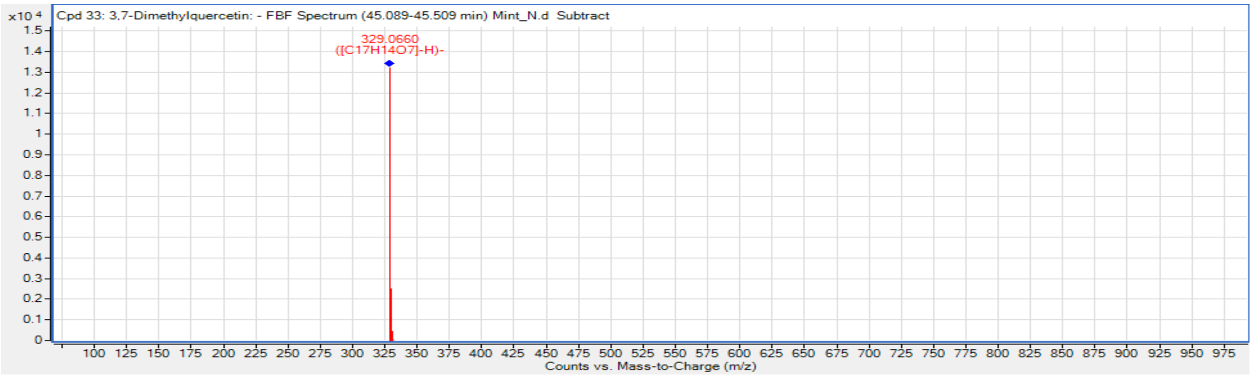

Compound 66

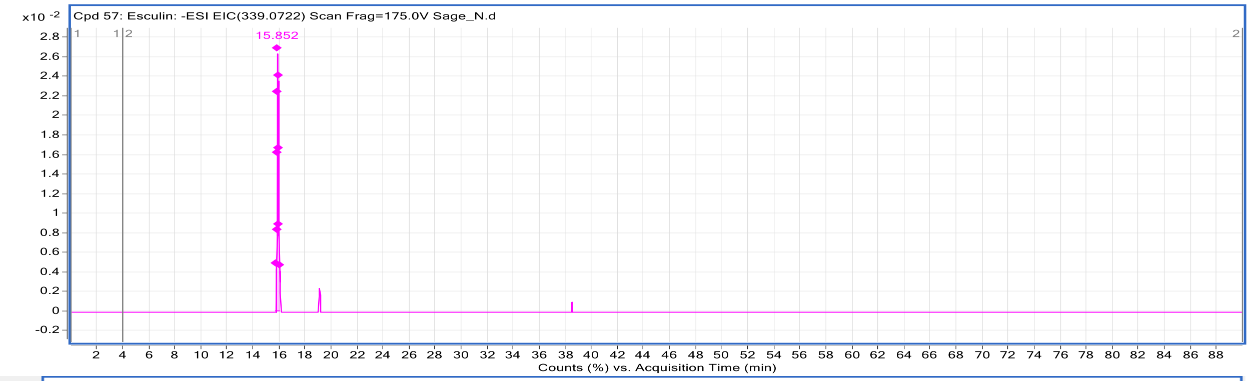

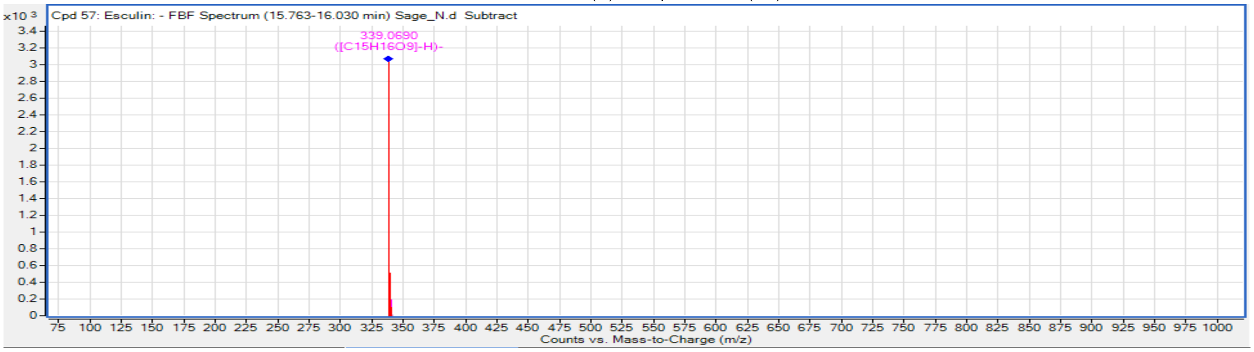

Compound 68

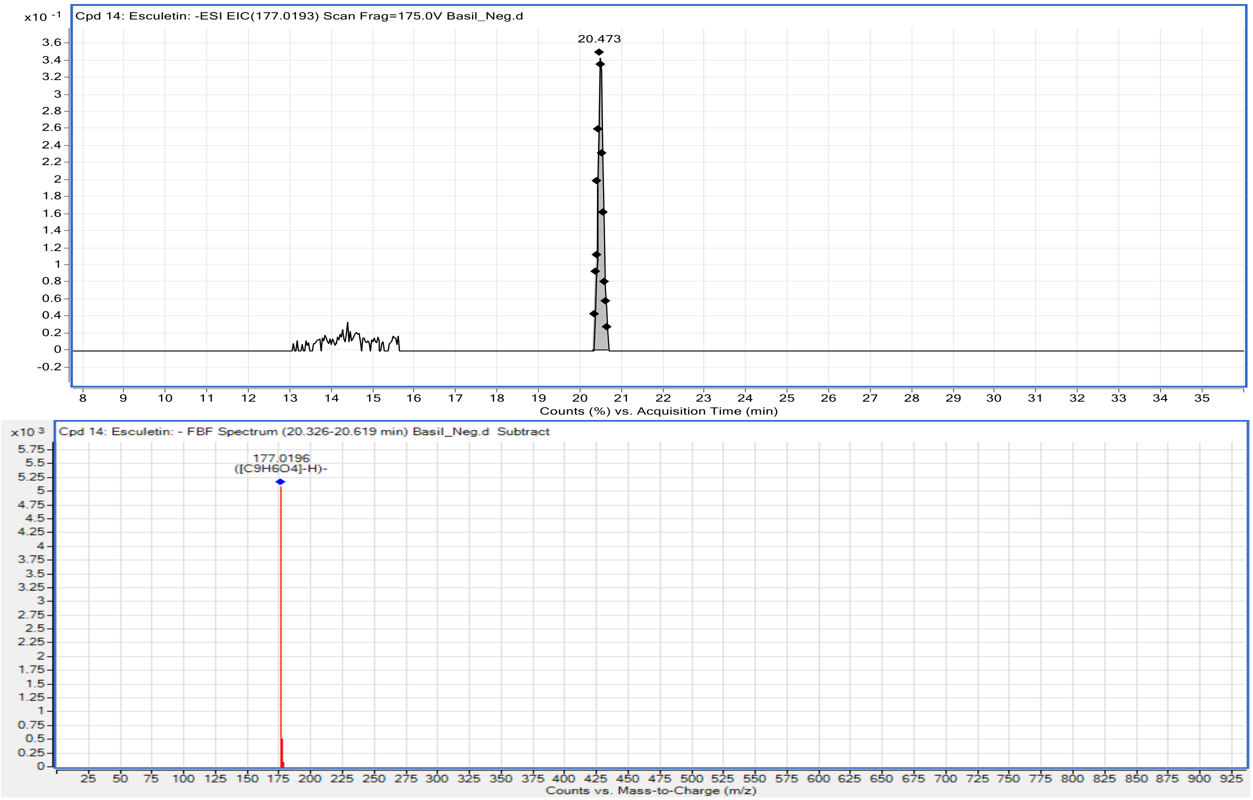

Compound 73

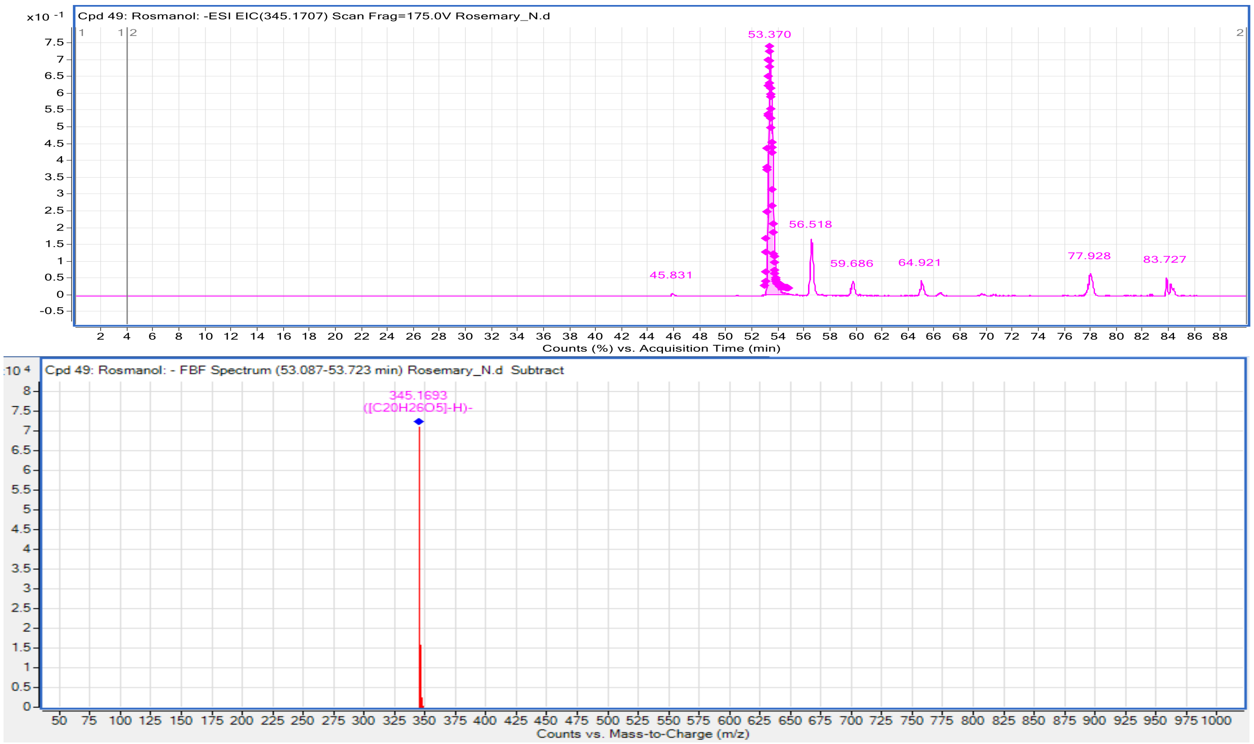

Compound 74

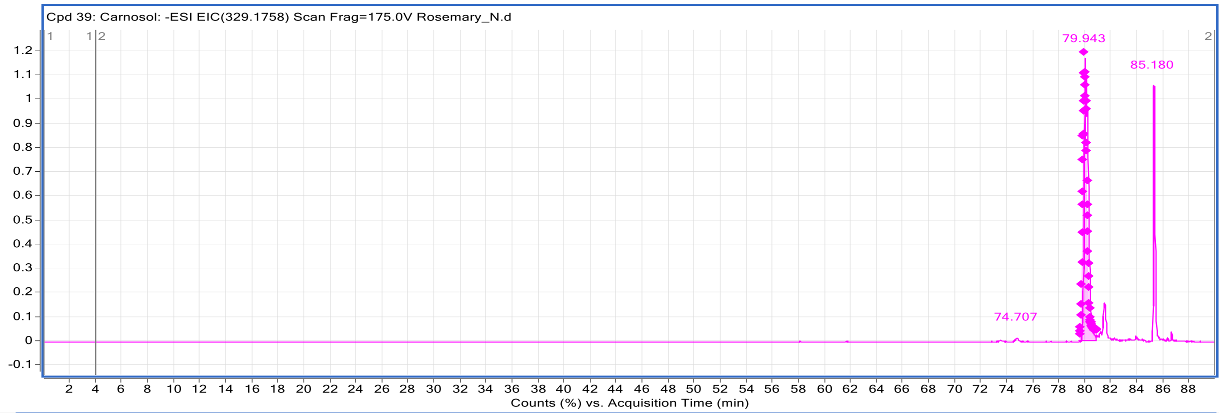

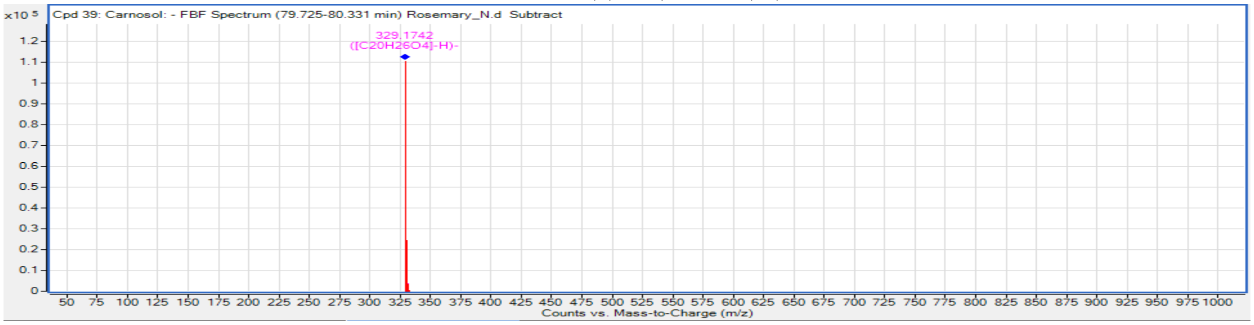

Compound 76

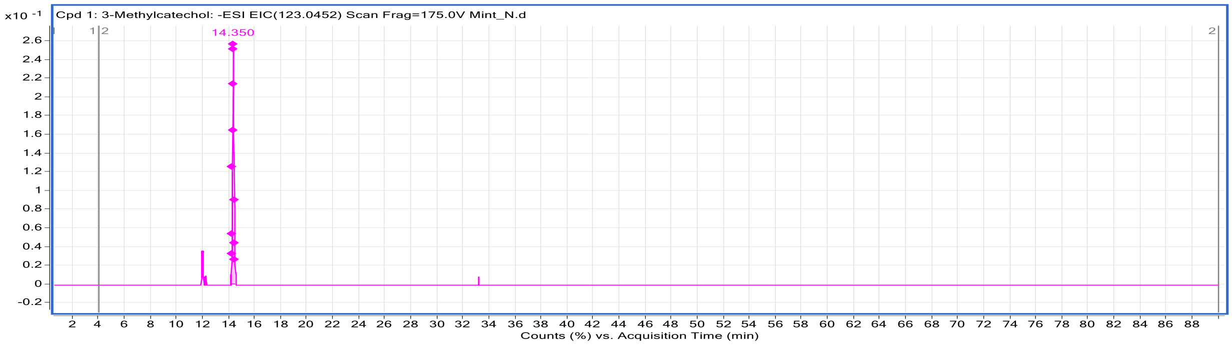

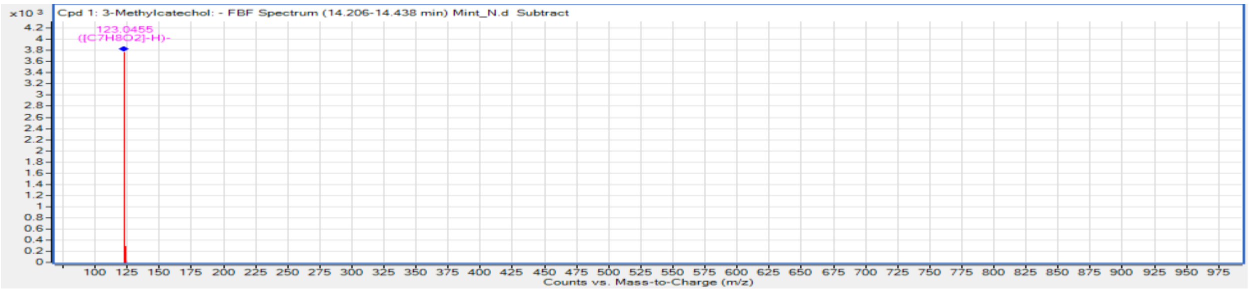

Compound 77

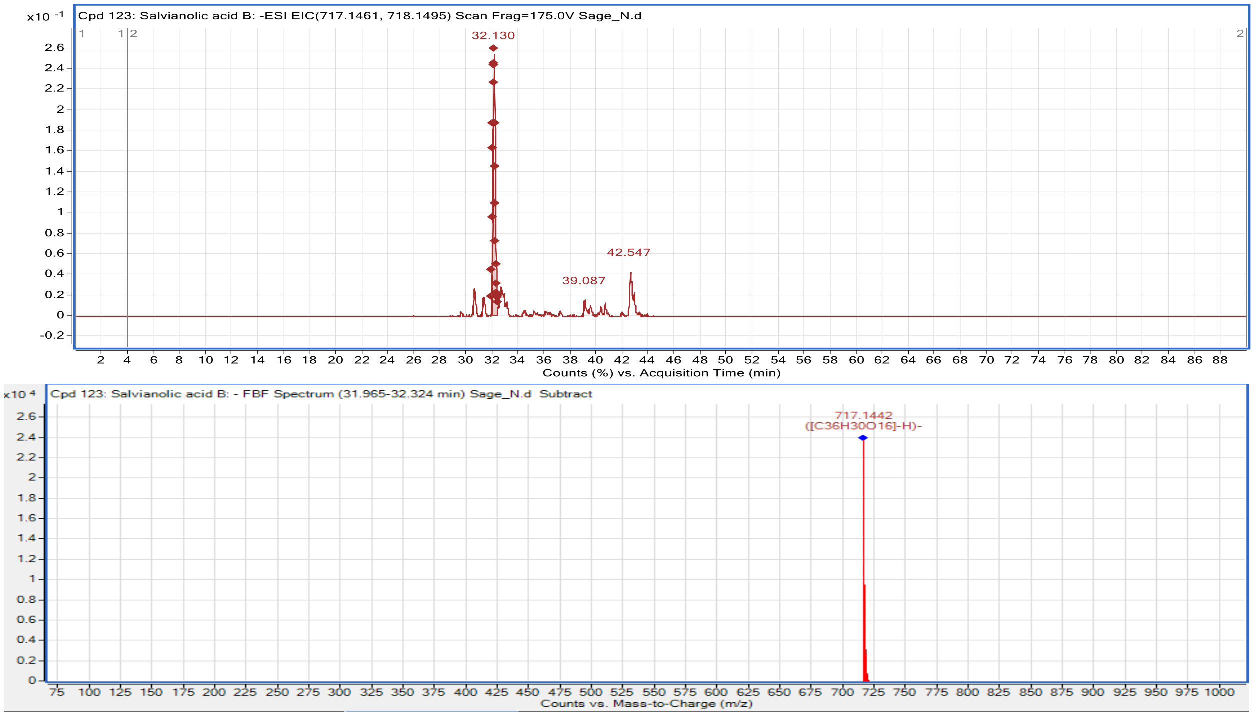

Figure S2: Chromatogram and spectrum of some selected compounds with their observed mass and retention time.

Table S1. HPLC-MS semi-quantification of abundant phenolic compounds in 10 herbs (µg/g)

**Compounds**	**Oregano**	**Mint**	**Thyme**	**Basil**	**Rosemary**	**Bay**	**Sage**	**Fenugreek**	**Dill**	**Parsley**
Gallic acid	57.02 ± 13.87	57.02 ± 11.31	59.59 ± 9.22	NQ	53.74 ± 7.92	56.63 ± 3.78	NQ	NQ	NQ	55.78 ± 6.54
Protocatechuic acid	331.45 ± 38.91	11.07 ± 0.99	NQ	273.79 ± 27.44	268.00 ± 18.11	NQ	269.83 ± 21.43	270.47 ± 24.61	282.58 ± 22.69	275.04 ±
Caftaric acid	NQ	NQ	81.89 ± 12.77	NQ	NQ	77.59 ± 9.56	NQ	79.42 ± 8.54	81.06 ± 12.49	NQ
p-hydroxybenzoic acid	162.12 ± 17.55	162.12 ± 23.44	75.95 ± 16.98	NQ	470.21 ± 39.32	NQ	NQ	NQ	NQ	NQ
Chlorogenic acid	122.20 ± 22.41	NQ	31.39 ± 12.89	117.51 ± 12.89	140.05 ± 23.56	106.00 ± 10.56	105.58 ± 12.54	109.56 ± 9.23	NQ	NQ
Caffeic acid	270.05 ± 19.18	NQ	32.12 ± 7.65	130.49 ± 11.54	129.26 ± 12.73	128.96 ± 15.24	129.29 ± 14.76	128.39 ± 11.54	NQ	NQ
Syringic acid	63.13 ± 11.08	163.13 ± 19.77	NQ	NQ	120.32 ±	77.39 ± 8.11	58.36 ± 8.12	58.12 ± 7.89	63.88 ± 7.65	59.70 ± 7.31
Epicatechin	116.69 ± 17.33	116.69 ± 12.44	14.15 ± 2.46	NQ	NQ	106.14 ± 9.36	NQ	110.74 ± 11.09	NQ	NQ
Coumaric acid	23.01 ± 0.91	NQ	NQ	NQ	340.44 ± 26.55	66.53 ± 5.67	58.62 ± 6.32	NQ	NQ	NQ
Ferulic acid	179.34 ± 33.01	NQ	NQ	NQ	124.13 ± 11.76	NQ	NQ	NQ	NQ	NQ
Sinapic acid	10.44 ± 0.89	NQ	NQ	30.11 ± 4.83	NQ	NQ	NQ	NQ	NQ	NQ
Rosmarinic acid	1650.13 ± 57.39	NQ	NQ	NQ	540.76 ± 27.31	NQ	NQ	NQ	NQ	NQ
Chicoric acid	71.57 ± 1.84	NQ	NQ	NQ	151.34 ± 11.43	NQ	NQ	NQ	NQ	NQ
Catechin	17.03 ± 1.01	13.17 ± 1.88	NQ	10.64 ± 1.83	NQ	14.58 ± 4.01	19.99 ± 2.11	24.60 ± 4.57	NQ	12.25 ± 3.61
Epicatechin gallate	NQ	NQ	36.67 ± 5.88	NQ	NQ	NQ	NQ	NQ	NQ	NQ
Quercetin-3-glucuronide	NQ	NQ	NQ	NQ	23.12 ± 2.89	14.86 ± 3.21	NQ	NQ	NQ	NQ
Quercetin-3-galactoside	NQ	NQ	NQ	NQ	NQ	NQ	NQ	NQ	NQ	NQ
Quercetin-3-glucoside	NQ	NQ	NQ	NQ	NQ	15.28 ± 3.03	14.65 ± 3.31	NQ	NQ	NQ
Kaempferol-3-glucoside	NQ	NQ	NQ	NQ	NQ	46.07 ± 4.31	NQ	NQ	NQ	NQ
Quercetin	93.44 ± 4.05	NQ	70.30 ± 10.43	NQ	170.95 ± 5.56	NQ	NQ	NQ	NQ	NQ
Luteolin 7-*O*-glucuronide	0.15 ±	NQ	NQ	NQ	2.13 ± 0.77	NQ	NQ	NQ	NQ	NQ
6-Hydroxyluteolin 7-*O*-rhamnoside	NQ	0.88 ± 0.02	NQ	NQ	1.07 ± 0.34	NQ	NQ	NQ	NQ	NQ
Myricetin 3-*O*-rhamnoside	NQ	NQ	NQ	0.55 ± 0.03	0.69 ± 0.47	NQ	NQ	NQ	NQ	NQ
Rosmanol	NQ	NQ	NQ	NQ	1.17 ± 0.23	NQ	NQ	NQ	NQ	NQ
Carnosol	NQ	NQ	NQ	NQ	0.11 ± 0.00	NQ	NQ	NQ	NQ	NQ
Carnosic acid	NQ	NQ	NQ	NQ	0.65 ± 0.01	NQ	NQ	NQ	NQ	NQ
Piceatannol	NQ	NQ	NQ	NQ	NQ	NQ	NQ	0.65 ± 0.08	NQ	NQ
Kaempferol	53.04 ± 7.11	NQ	25.04 ± 3.23	26.95 ± 2.48	81.55 ± 7.34	NQ	35.68 ± 4.67	NQ	NQ	NQ

*NQ = Not quantified
